# Molecular dissection of an intronic enhancer governing cold-induced expression of the vacuolar invertase gene in potato

**DOI:** 10.1093/plcell/koae050

**Published:** 2024-02-20

**Authors:** Xiaobiao Zhu, Airu Chen, Nathaniel M Butler, Zixian Zeng, Haoyang Xin, Lixia Wang, Zhaoyan Lv, Dani Eshel, David S Douches, Jiming Jiang

**Affiliations:** Anhui Province Key Laboratory of Horticultural Crop Quality Biology, School of Horticulture, Anhui Agricultural University, Hefei 230036, Anhui Province, China; Department of Horticulture, University of Wisconsin-Madison, Madison, WI 53706, USA; Anhui Province Key Laboratory of Horticultural Crop Quality Biology, School of Horticulture, Anhui Agricultural University, Hefei 230036, Anhui Province, China; Department of Horticulture, University of Wisconsin-Madison, Madison, WI 53706, USA; Vegetable Crops Research Unit, United States Department of Agriculture-Agricultural Research Service, Madison, WI 53706, USA; Department of Horticulture, University of Wisconsin-Madison, Madison, WI 53706, USA; Department of Biological Science, College of Life Sciences, Sichuan Normal University, Chengdu 610101, Sichuan Province, China; Plant Functional Genomics and Bioinformatics Research Center, Sichuan Normal University, Chengdu 610101, Sichuan Province, China; Department of Plant Biology, Michigan State University, East Lansing, MI 48824, USA; Anhui Province Key Laboratory of Horticultural Crop Quality Biology, School of Horticulture, Anhui Agricultural University, Hefei 230036, Anhui Province, China; Anhui Province Key Laboratory of Horticultural Crop Quality Biology, School of Horticulture, Anhui Agricultural University, Hefei 230036, Anhui Province, China; Department of Postharvest Science, The Volcani Institute, ARO, Rishon LeZion 50250, Israel; Department of Plant, Soil, and Microbial Sciences, Michigan State University, East Lansing, MI 48824, USA; Michigan State University AgBioResearch, East Lansing, MI 48824, USA; Department of Horticulture, University of Wisconsin-Madison, Madison, WI 53706, USA; Department of Plant Biology, Michigan State University, East Lansing, MI 48824, USA; Michigan State University AgBioResearch, East Lansing, MI 48824, USA; Department of Horticulture, Michigan State University, East Lansing, MI 48824, USA

## Abstract

Potato (*Solanum tuberosum*) is the third most important food crop in the world. Potato tubers must be stored at cold temperatures to minimize sprouting and losses due to disease. However, cold temperatures strongly induce the expression of the potato vacuolar invertase gene (*VInv*) and cause reducing sugar accumulation. This process, referred to as “cold-induced sweetening,” is a major postharvest problem for the potato industry. We discovered that the cold-induced expression of *VInv* is controlled by a 200 bp enhancer, *VInv*In2En, located in its second intron. We identified several DNA motifs in *VInv*In2En that bind transcription factors involved in the plant cold stress response. Mutation of these DNA motifs abolished *VInv*In2En function as a transcriptional enhancer. We developed *VInv*In2En deletion lines in both diploid and tetraploid potato using clustered regularly interspaced short palindromic repeat (CRISPR)/CRISPR-associated nuclease 9 (Cas9)-mediated gene editing. *VInv* transcription in cold-stored tubers was significantly reduced in the deletion lines. Interestingly, the *VInv*In2En sequence is highly conserved among distantly related *Solanum* species, including tomato (*Solanum lycopersicum*) and other non-tuber-bearing species. We conclude that the *VInv* gene and the *VInv*In2En enhancer have adopted distinct roles in the cold stress response in tubers of tuber-bearing *Solanum* species.

## Teaser

An intronic enhancer is responsible for cold-induced expression of the vacuolar invertase gene in potato.

## Introduction

Potato (*Solanum tuberosum*) is the third most important food crop in the world in terms of human consumption (Devaux *et al*. 2020). In addition, French fries and potato chips are among the most consumed snacks, especially in developed countries. Unlike the grain crops, storage is one of the most important issues related to the potato industry because tubers must be stored at cold temperatures to prevent sprouting and diseases. Unfortunately, cold storage triggers the breakdown of starch and accumulation of reducing sugars, which is referred to as “cold-induced sweetening” (CIS) (Dale and Bradshaw 2003), a costly and nagging problem for the potato processing industry (Sowokinos 2001). The reducing sugars in tubers will react with free amino acids via a nonenzymatic, Maillard-type reaction during high-temperature processing. This reaction results in products with dark color and bitter taste and produces acrylamide, a potential carcinogen (Mottram *et al*. 2002; Stadler *et al*. 2002). Reducing sugars are the primary determinants for the acrylamide content in fried potato products (Amrein *et al*. 2003; Becalski *et al*. 2004; Zhu *et al*. 2016). Thus, developing methods to minimize reducing sugars in cold-stored tubers has been an important research focus to reduce acrylamide in fried potato products.

CIS was reported to be associated with numerous genetic loci based on genetic mapping (Menendez *et al*. 2002; Li *et al*. 2008; Braun *et al*. 2017), genome-wide association studies (GWAS) (Byrne *et al*. 2020), and comparative proteomics studies between CIS-resistant and CIS-susceptible potato cultivars (Fischer *et al*. 2013). This can be explained by the fact that CIS is likely linked to numerous enzymes that function in central carbohydrate metabolism in potato tubers (Sowokinos 2001). The vacuolar invertase gene (*VInv*) received a major attention on its potential role in CIS. Partial control of CIS was accomplished by manipulating the activity of the VINV protein (Greiner *et al*. 1999; Agarwal *et al*. 2003) or the transcription of the *VInv* gene (Zrenner *et al*. 1996; Zhang *et al*. 2008). Silencing of *VInv* using RNAi resulted in nearly full control of CIS in at least some potato cultivars (Bhaskar *et al*. 2010; Ye *et al*. 2010). Interestingly, *VInv* gene transcription in tubers is maintained at a minimal level under room temperature. *VInv* is dramatically upregulated during cold storage in CIS-susceptible potato cultivars (Zrenner *et al*. 1996; Bagnaresi *et al*. 2008; Bhaskar *et al*. 2010), causing rapid accumulation of reducing sugars. Silencing of the *VInv* gene has been proven to be an effective approach to control CIS in many potato cultivars (Bhaskar *et al*. 2010; Ye *et al*. 2010; Liu *et al*. 2011; Wu *et al*. 2011; Clasen *et al*. 2016; Ly *et al*. 2023). Concordantly, overexpression of *StInvInh2*, which encodes a vacuolar invertase inhibitor, can also reduce potato CIS (Liu *et al*. 2013; Mckenzie *et al*. 2013).

Interestingly, the upregulation of *VInv* in cold-stored tubers is not controlled by its promoter (Ou *et al*. 2013). The *VInv* promoter is required to respond to sugars, indole-3-acetic acid (IAA), and gibberellic acid (GA_3_) but not to cold temperatures (Ou *et al*. 2013). Here, we report the discovery of a 200 bp transcriptional enhancer, *VInv*In2En, located in the second intron of *VInv*. This enhancer is responsible for the cold-induced expression of the *VInv* gene. We identified several DNA motifs that bind transcription factors (TFs) involved in plant response to cold stress. Mutation of these motifs abolished the function of *VInv*In2En. We developed *VInv*In2En deletion lines in both diploid and tetraploid potato lines using clustered regularly interspaced short palindromic repeat (CRISPR)/CRISPR-associated nuclease 9 (Cas9)-mediated genome editing. *VInv* transcription was significantly reduced in the deletion lines during cold storage. Interestingly, the *VInv*In2En sequence was found to be highly conserved among distantly related plant species, revealing an evolutionary trajectory of the *VInv* gene in response to cold stress in the tuber-bearing *Solanum* species.

## Results

### Discovery of a cold-responsive intronic enhancer within *VInv* gene

**Figure 1.**
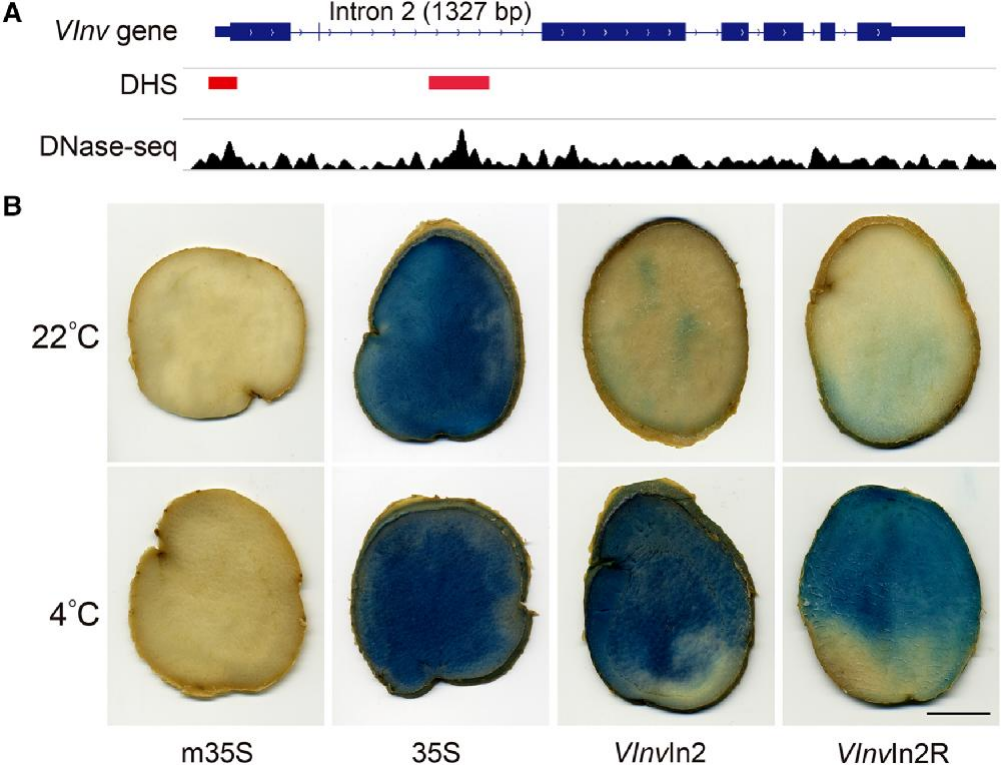
Discovery of a cold-responsive intronic enhancer in *VInv* gene. **A)** DHSs associated with *VInv* gene. DHS map was developed from tuber tissue of DM1-3 potato. Two DHSs, 1 at the 5′ of the gene and 1 in the second intron, were detected. **B)** GUS reporter gene assays of the second intron of *VInv* gene in ‘Katahdin’ potato. Constructs using a minimal 35S promoter (m35S) and a full-length 35S promoter were used as negative and positive controls. Tubers from transgenic ‘Katahdin’ lines developed using the intronic construct (*VInv*In2) and a reverse construct (*VInv*In2R) showed minimal GUS signals under room temperature (22 °C). Strong GUS signals were detected from tubers after 4 wks of cold storage under 4 °C. The scale bar represents 2 cm.

Genomic regions containing active *cis*-regulatory elements (CREs), such as promoters and transcriptional enhancers, can be identified as DNase I hypersensitive sites (DHSs) (Zhang *et al*. 2012; Jiang 2015; Zhao *et al*. 2018). We previously developed genome-wide DHS maps in DM1-3 potato using chromatin isolated from tuber tissue (Zeng *et al*. 2019). DM1-3 is a homozygous diploid clone and was developed from chromosome doubling of a monoploid derived from an *S. tuberosum* Phureja Group clone (Mribu and Veilleux 1990; Paz and Veilleux 1999) and has been fully sequenced (2*n* = 2*x* = 24) (The Potato Genome Sequencing Consortium 2011; Pham *et al*. 2020). We detected a 475 bp DHS within the second intron of *VInv* (Fig. 1A), suggesting that this intron may play a role in the regulation of the expression of *VInv*. We have recently demonstrated the enhancer function of several intronic DHSs in Arabidopsis (*Arabidopsis thaliana*) (Meng *et al*. 2021).

To confirm its *cis*-regulatory function, we cloned the entire *VInv* second intron (1,327 bp) from RH potato, which is a heterozygous diploid clone (van Os *et al*. 2006) and has recently been fully sequenced (Zhou *et al*. 2020). RH is susceptible to CIS (Supplementary Fig. S1). The intron was cloned into the pKGWFS 7.0 vector containing a minimal 35S promoter (−50 to −2 bp) (m35S) and the β-glucuronidase (GUS) reporter gene (Zhu *et al*. 2015). ‘Katahdin,’ a CIS-susceptible tetraploid cultivar (Bhaskar *et al*. 2010), was used for transformation. We developed 20 transgenic ‘Katahdin’ lines from the intron construct (*VInv*In2) and 20 lines from a reverse construct (*VInv*In2R) in which the sequence orientation of the cloned intron is reversed. We detected minimal GUS signals in transgenic tubers stored at room temperature (22 °C). In contrast, substantially enhanced GUS signals were detected in the transgenic tubers after 4 wks of cold storage (4 °C) (Fig. 1B). These results indicate that intron 2 of *VInv* contains an enhancer that is responsible for its cold-induced expression.

### Dissection of intronic enhancer via reporter gene assays in *A. thaliana*

**Figure 2.**
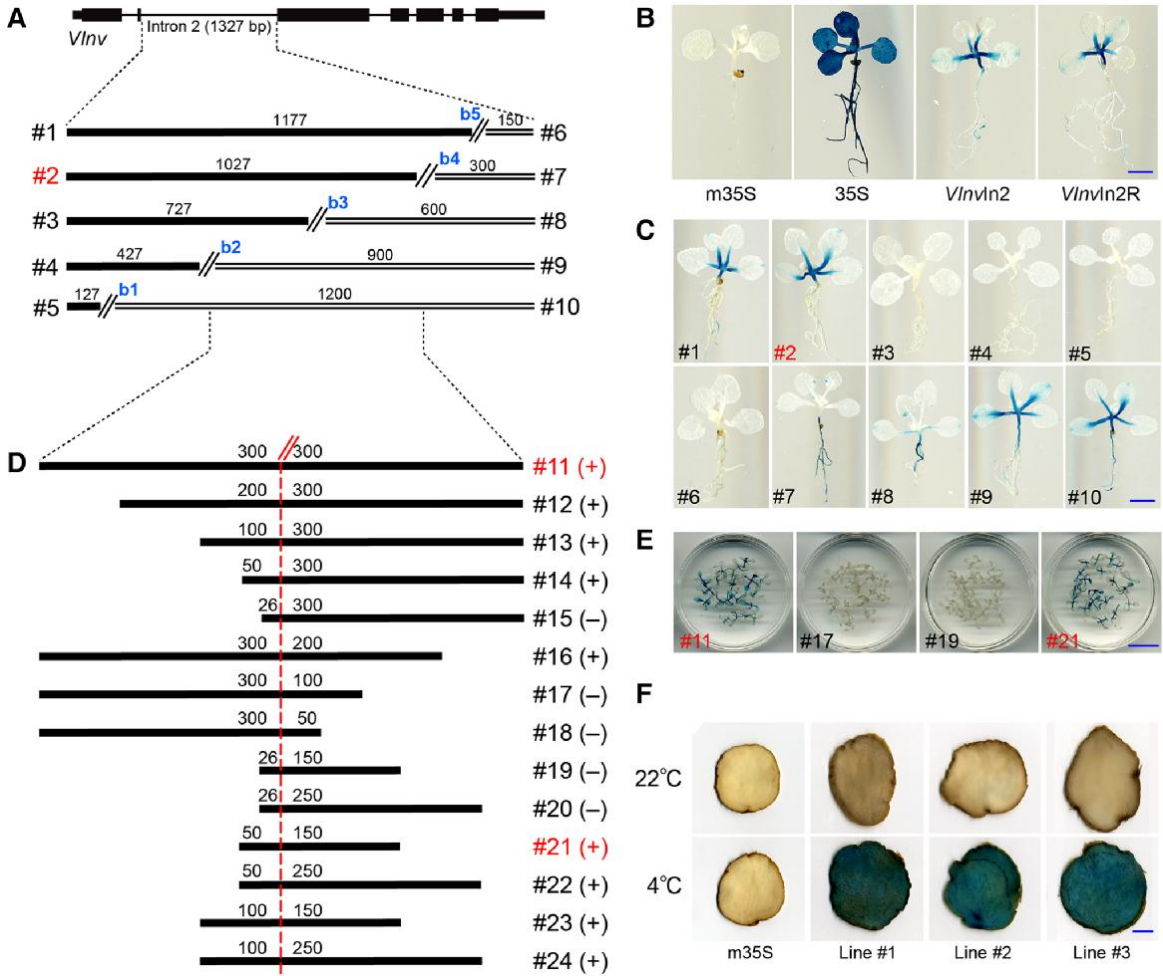
Identification of transcriptional enhancers in intron 2 of *VInv* gene. **A)** A diagram illustrating the sizes and positions of 10 subfragments derived from intron 2 of the *VInv* gene. The 1,327 bp intron was divided into 10 fragments (#1 to #10) using 5 breaks (b1 to b5). **B)** GUS reporter gene assays of intron 2 in *A. thaliana*. Constructs with minimal 35S promoter (m35S) and a full-length 35S promoter (35S) were used as negative and positive controls. **C)***GUS* expression patterns of representative *A. thaliana* transgenic seedlings derived from each of the 10 constructs consisting of a fragment ligated with the m35S promoter and the GUS reporter gene. **D)** A diagram illustrating the sizes and positions of the 13 fragments derived from the DNA fragment #11. A dashed red line marks the middle point of the 600 bp segment #11. “+” and “−” indicate the derived transgenic seedlings showing positive and negative GUS signals, respectively. **E)** GUS staining of 20 *A. thaliana* transgenic seedlings derived from constructs #11, #17, #19, and #21, respectively. **F)** GUS reporter gene assay of the 200 bp *VInv*In2En enhancer in ‘Katahdin’ potato. Tubers from 3 independent transgenic lines showed minimal GUS signals under 22 °C but strong signals from tubers after 4 wks of cold storage under 4 °C. All numbers above bars/lines in **A)** and **D)** indicate base pairs. The scale bar represents 2 mm in **B**, **C)** and 1 cm in **E**, **F)**. Fragment numbers highlighted in red color in **A)**, **C)**, **D)**, and **E)** indicate representative constructs with full enhancer function.

Since the entire intron 2 from RH potato was used for GUS reporter assays, the precise size and position of the predicted enhancer within intron 2 could not be determined. We attempted to fine-map the enhancer using reporter gene assay in *A. thaliana*. We first examined the GUS signal profiles of transgenic *A. thaliana* plants using the *VInv*In2 and *VInv*In2R constructs. Consistent and strong GUS signals were detected in stems and petioles in transgenic plants derived from both constructs. In addition, relatively weak and sporadic GUS signals were also detected in roots (Fig. 2B). The *VInv* gene is expressed at relatively high levels in several nontuber tissues of potato, including both petiole and stem (The Potato Genome Sequencing Consortium 2011; Zhou *et al*. 2020). Thus, the GUS signal patterns observed in the transgenic *A. thaliana* plants correspond well with the *VInv* expression patterns in potato tissues.

We next divided the 1,327 bp intron 2 into 10 DNA fragments (#1 to #10) using 5 breaks (b1 to b5; Fig. 2A). Each fragment was ligated to the m35S promoter and cloned into the pKGWFS 7.0 vector. Transgenic plants derived from DNA fragments #1, #2, #9, and #10 showed strong GUS signals in stems and petioles, which were similar to the transgenic plants developed from the *VInv*In2 and *VInv*In2R constructs. Similar but weaker signals were detected from transgenic plants derived from fragment #8 (Fig. 2C). These results indicated that the enhancer driving *GUS* expression in stems and petioles is located between b2 and b4, which was named fragment #11 (Fig. 2D).

We next further divided the 600 bp fragment #11 into 13 subfragments (#12 to #24; Fig. 2D) for GUS reporter assays. Transgenic plants derived from construct #21 (200 bp) showed strong GUS signals in stems and petioles. In contrast, transgenic plants derived from constructs #17 and #19 did not show GUS signals (Fig. 2E). Thus, the core enhancer in intron 2 was mapped within the 200 bp #21 sequence and was named as *VInv*In2En thereafter. *VInv*In2En spans 678 to 877 bp in the intron and is located within the 475 bp DHS, which spans 597 to 1,071 bp in the intron (Fig. 1A).

To confirm the function of *VInv*In2En in potato, we developed transgenic lines using a *VInv*In2En-m35S-GUS construct in ‘Katahdin’ potato. We detected minimal GUS signals in tubers stored at room temperature (22 °C) but strong GUS signals in cold-stored tubers (4 °C) from 3 independent transgenic lines (Fig. 2F). Thus, the *VInv*In2En sequence retains the same function as the entire intron 2 in potato (Fig. 1B).

### Identification of DNA motifs related to *VInv*In2En function

**Figure 3.**
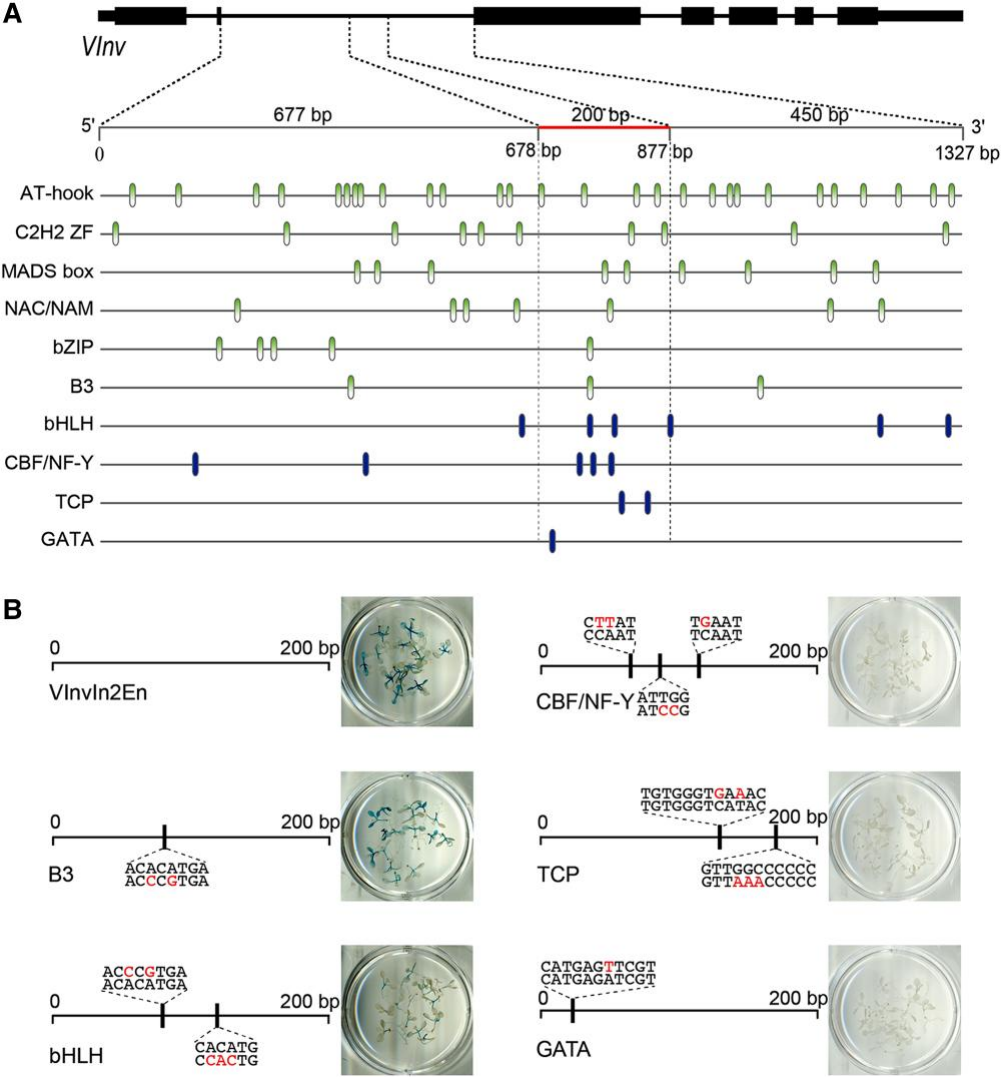
Distribution and function of DNA motifs in intron 2 and the *VInv*In2En enhancer. **A)** Distribution of DNA motifs related to TFs involved in response to cold stress. Each vertical bar represents a potential TF-binding site. A red horizontal bar marks the position of the 200 bp enhancer. Vertical blue bars indicate that the binding sites of a relevant TF are enriched or exclusively located within the 200 bp enhancer. Vertical green bars indicate that the binding sites of a relevant TF are not enriched within the enhancer. **B)** Transgenic assays of *VInv*In2En with mutated DNA motifs related to 5 different TFs. Red-colored nucleotides indicate the replaced sequence(s) in each construct. No GUS signals were detected in any transgenic *A. thaliana* plants derived from the 3 constructs with mutated motifs related to CBF/NF-Y, TCP, and GATA.

We speculated that *VInv*In2En contains DNA motifs bound by TFs involved in plant response to cold stress. We identified putative DNA motifs related to a total of 15 TFs in the intron 2 sequence of RH (Zhou *et al*. 2020). These motifs were consistently detected by 2 independent programs using both CIS-BP (Weirauch *et al*. 2014) and PlantPAN 3.0 (Chow *et al*. 2019). Interestingly, 10 of these 15 TFs were previously reported to be associated with responses to cold stress in 1 or multiple plant species (Fig. 3A), including AT-hook (Dahro *et al*. 2022), C2H2 ZF (He *et al*. 2019), MADS-box (Chen *et al*. 2019), NAC/NAM (Li *et al*. 2016), bHLH (Xie *et al*. 2012), CBF/NF-Y (Zhou *et al*. 2022; Zhang *et al*. 2023), bZIP (Liu *et al*. 2018; Li *et al*. 2022b), B3 (Verma and Bhatia 2019), TCP (Li *et al*. 2022a), and GATA (Zhang *et al*. 2021).

Several TF motifs were enriched in the 200 bp *VInv*In2En, including bHLH and CBF/NF-Y. In addition, motifs related to TCP and GATA were found only in the 200 bp enhancer region (Fig. 3A). We designed mutated versions of *VInv*In2En to test the function of the DNA motifs related to B3, bHLH, CBF/NF-Y, TCP, and GATA. In each construct, the target motif(s) were mutated by replacing 1 to 3 nucleotide(s) within the sequence (Fig. 3B, Supplementary Table S1). Transgenic *A. thaliana* plants using *VInv*In2En with a mutated B3 motif showed similar GUS signal patterns as those from wild-type (WT) *VInv*In2En. Reduced GUS signals were detected from transgenic plants using *VInv*In2En with 2 mutated bHLH motifs. In contrast, we did not detect any GUS signals from transgenic plants derived from the 3 constructs with mutated motifs related to CBF/NF-Y, TCP, and GATA. Most strikingly, a single-nucleotide mutation within the GATA motif resulted in a complete loss of function of the *VInv*In2En enhancer (Fig. 3B). These results indicated that CBF/NF-Y, TCP, and GATA all play important roles for *VInv*In2En driving *GUS* expression in stems and petioles.

**Figure 4.**
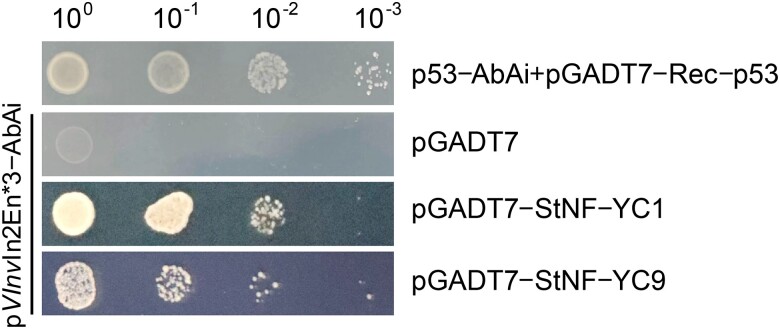
Identification of StNF-YC1 and StNF-YC9 proteins that bind to *VInv*In2En using Y1H assay. Triple copies of the *VInv*In2En sequence (*VInv*In2En*3) were synthesized to develop the bait plasmid p*VInv*In2En*3-AbAi. The pGADT7 vector was used as negative control, and a combination of 2 constructs (p53-AbAi and pGADT7-Rec-p53) was used as positive control.

To seek additional functional evidence of the 3 DNA motifs identified in *VInv*In2En, we conducted a yeast 1-hybrid (Y1H) assay using triple copies of the *VInv*In2En sequence as a bait (see Materials and methods). A total of 387 yeast colonies were obtained by screening a cDNA library developed from cold-treated tuber tissues from RH potato. All 387 clones were fully sequenced. The sequences were used for BLAST search in the DM1-3 potato cDNA (v6.1) database using Spud DB BLASTn program (http://spuddb.uga.edu/blast.shtml). All of the best matched cDNA sequences with an *E*-value < 1e^−5^ and a minimum sequence identity of 82% were kept for further analyses. A total of 33 unique cDNA sequences were obtained after filtering out repeated cDNA sequences. The candidate proteins related to these 33 cDNAs were used for further validation using point-to-point Y1H assay (see Materials and methods). Five proteins, including StNF-YC1 and StNF-YC9 (Fig. 4), were validated as positive interacting proteins binding to *VInv*In2En. The StNF-YC1 and StNF-YC9 proteins share 86% and 73% sequence similarity with AtNF-YC1 and AtNF-YC9 of *A. thaliana*, respectively. These results validated the predicted role of CBF/NF-Y family TFs in the regulation of CIS mediated by the *VInv*In2En enhancer.

### Genome editing of *VInv*In2En in diploid potato

**Figure 5.**
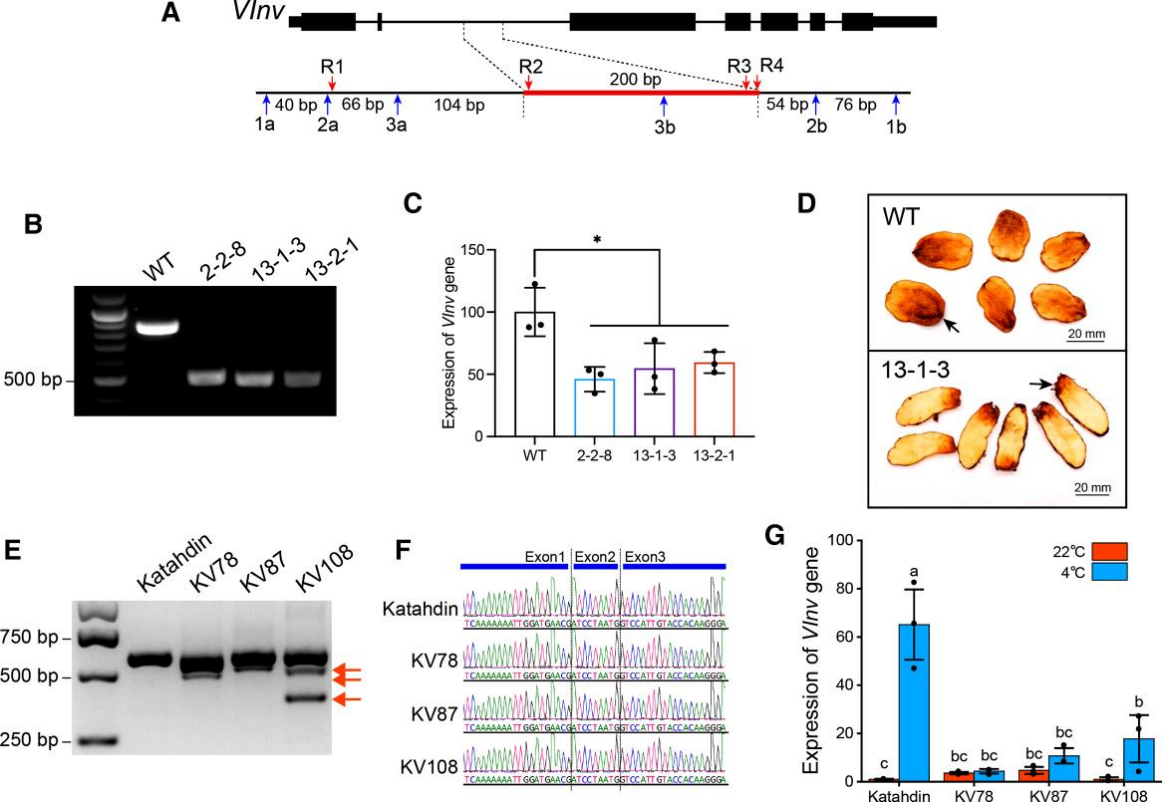
Functional validation of the *VInv*In2En enhancer using genome editing. **A)** A diagram illustrating the positions of all sgRNAs within and outside of intron 2 of *VInv* gene. The red bar marks the 200 bp enhancer *VInv*In2En. Red arrows indicate the position of sgRNAs R1, R2, R3, and R4. Blue arrows indicate the position of sgRNAs 1a, 2a, 3a, 1b, 2b, and 3b. **B)** Gel electrophoresis of PCR products amplified from the 3 homozygous CRISPR/Cas9 deletion lines (2-2-8, 13-1-3, and 13-2-1) developed from the WT DMF5-73-1. **C)** RT-qPCR-based transcription analysis of *VInv* gene in cold-stored potato tissues from the 3 homozygous deletion lines (2-2-8, 13-1-3, and 13-2-1). All 3 lines showed significant reduction of *VInv* expression relative to the *Actin97* reference gene. The *y* axis represents the relative expression level normalized by setting *VInv* expression in cold-stored tubers of the WT DMF5-73-1 to 1. Data are presented as mean ± Sd from 3 biological replicates and were tested by the Student *t* test (*P < 0.05). **D)** Chipping of tubers from deletion line 13-1-3 and from the WT DMF5-73-1. Note: (i) the dark color toward 1 end of each chip is caused by the “jelly end” problem (2 examples are indicated by arrows) associated with both 13-1-3 and WT. (ii) 13-1-3 is a selfed progeny of a T0 DMF5-73-1 (heterozygous) transgenic line. Thus, the tubers from the 2 lines show different shapes. Three tubers from each line were used for chipping, and 2 chips from each tuber were included in the illustration. **E)** Gel electrophoresis of PCR products amplified from the genomic DNA of 3 T0 CRISPR/Cas9 lines (KV78, KV87, and KV108) developed from tetraploid potato cultivar ‘Katahdin.’ Red arrows indicate fragments resulted from deletions within *VInv*In2En. **F)** Sequencing of PCR products amplified from cDNAs of the 3 CRISPR/Cas9 lines. Normal splicing between exons 1 and 3 was detected in all 3 lines. **G)** RT-qPCR-based analysis of *VInv* expression relative to the *Actin97* gene of the 3 CRISPR/Cas lines. Expression was analyzed using tubers after 2 wks of storage at 22 °C and 4 °C, respectively. The *y* axis represents the relative expression level normalized by setting *VInv* expression in 22 °C-stored tubers of the WT ‘Katahdin’ to 1. Data are presented as mean ± Sd from 3 biological replicates and were tested by using PROC GLM ANOVA. Different lower case letters represent statistically significant differences at *P* = 0.05.

The in vivo function of a predicted enhancer can be validated by mutation or deletion using genome editing (Meng *et al*. 2021; Zhao *et al*. 2022; Fang *et al*. 2023). We attempted to develop *VInv*In2En deletion lines in potato to validate its in vivo function. We first conducted CRISPR/Cas experiments using a self-compatible diploid clone DMF5-73-1. This clone was self-pollinated for 5 generations from a self-compatible diploid hybrid DM1-3 × M6 (Endelman and Jansky 2016). DMF5-73-1 is amenable to *Agrobacterium*-mediated transformation (Butler *et al*. 2020). Five sgRNAs flanking *VInv*In2En (1a, 2a, 3a, 1b, and 2b) and a single sgRNA (3b) targeting *VInv*In2En (Fig. 5A, Supplementary Table S2) were designed and assembled into a single construct (Supplementary Fig. S2). Primary transformants were generated using a hairy root-based procedure to create stable CRISPR/Cas mutants in the first generation (T0) (Butler *et al*. 2020). T0 events carrying targeted deletions were self-pollinated, and the progeny were screened for homozygous mutations (T1). We identified 3 homozygous T1 deletion lines (Fig. 5B). Two lines, 13-1-3 and 13-2-1, were derived from the same hairy root culture. Sequencing analysis showed that deletion line 2-2-8 lost 369 bp, including the entire 200 bp *VInv*In2En. Lines 13-1-3 and 13-2-1 lost 394 bp, including the first 122 bp of *VInv*In2En (Supplementary Fig. S3), which spans the GATA and the 3 CBF/NF-Y motifs.

DMF5-73-1 is not susceptible to CIS and expresses a weak CIS phenotype. Tubers harvested from the 3 deletion lines were stored at 12.8 °C for 6 wks followed by at 6.7 °C for 9 additional wks, a storage procedure used to maximize the CIS phenotype. Tuber tissues were then sampled for RNA extraction and RT-qPCR analysis. We found that the expression of *VInv* gene was reduced by 54% for 2-2-8, 45% for 13-1-3, and 41% for 13-2-1, respectively, compared with the WT DMF5-73-1 (Fig. 5C). However, it was challenging to perform chipping analysis from the deletion lines because all 3 lines have small tubers and are associated with the “jelly end” defect derived from the parental clone DM1-3 (Endelman and Jansky 2016) (Fig. 5D). Nevertheless, potato chips processed from cold-stored tubers of line 13-1-3 showed a lighter color compared with those processed from WT DMF5-73-1 (Fig. 5D).

### Genome editing of *VInv*In2En in tetraploid potato

DMF5-73-1 is ideal for CRISPR/Cas experiments due to its self-compatibility that allows for identification of homozygous deletions. However, DMF5-73-1 is not an ideal line to accurately evaluate the impact of *VInv*In2En on CIS since it is resistant to CIS and has poor tuber traits. In addition, DMF5-73-1 retains a significant level of heterozygosity. Hence, the homozygous deletion lines developed from this clone are phenotypically different from the parental DMF5-73-1 (Fig. 5D). We next attempted to conduct CRISPR/Cas experiments in ‘Katahdin,’ a tetraploid potato cultivar that is highly susceptible to CIS (Bhaskar *et al*. 2010). We first amplified and sequenced intron 2 of *VInv* from ‘Katahdin.’ We identified 3 haplotypes: A (2 copies), B, and C. These haplotypes are differentiated by SNPs and small indels, including those within the *VInv*In2En region (Supplementary Fig. S4). We designed 4 sgRNAs, including R1 outside of *VInv*In2En and R2, R3, and R4 inside the *VInv*In2En boundary (Fig. 5A, Supplementary Fig. S4 and Table S2). The 4 sgRNAs were assembled into a single construct for CRISPR/Cas experiments (Supplementary Fig. S5).

We identified 3 different T0 CRISPR/Cas9 lines, KV78, KV87, and KV108. PCR amplifications using primers *VInv*-Edit-F/R that span the 4 sgRNAs (Supplementary Fig. S4 and Table S3) produced additional smaller bands as well as the WT band (Fig. 5E), suggesting that all 3 T0 lines contain both intact and deleted intron 2, possibly derived from different lineages of cells. We then isolated and mixed all DNA fragments visible on the agarose gel, including the WT band, from all 3 lines. The mixed DNA fragments were cloned, and a minimum of 60 randomly selected clones from each line were fully sequenced. Sequence analysis confirmed that each of the 3 T0 lines contained different types of deletions within *VInv*In2En, ranging from 3 to 124 bp deletions within *VInv*In2En associated with haplotype A and 1 to 15 bp deletions within *VInv*In2En associated with haplotype B (Supplementary Fig. S6). However, no deletions were detected in *VInv*In2En associated with haplotype C, probably due to the SNPs located in the PAM sequences downstream of sgRNAs R2 and R3. Based on the number of individual sequences related to *VInv*In2En, 67.7% of the haplotype A sequences from KV78 contained a deletion ranging from 4 to 97 bp; 64.7% of the haplotype A sequences from KV87 contained a deletion of 3 to 64 bp; 59.3% of the haplotype A sequences from KV108 contained a 4 to 124 bp deletion (Supplementary Fig. S6).

We amplified the cDNAs of *VInv* from the 3 CRISPR/Cas lines using primers Splicing-F/R spanning exons 1 to 3 (Supplementary Table S3). Sequencing of the PCR products showed that the transcripts from the 3 CRISPR/Cas lines were identical to those from WT ‘Katahdin’ (Fig. 5F). Thus, the deletions occurred in *VInv*In2En did not affect the splicing of the *VInv* gene. We next analyzed the expression of the *VInv* gene in the 3 CRISPR/Cas lines using RNAs isolated from tubers stored for 2 wks under 22 °C and 4 °C, respectively. A similar and minimal level of *VInv* expression was observed in 22 °C-stored tubers from WT ‘Katahdin’ and all 3 CRISPR/Case lines. In contrast, the expression level of *VInv* in 4 °C-stored tubers of the 3 CRISPR/Cas lines was only 6.6%, 16.4%, and 27.3%, respectively, of the WT ‘Katahdin’ (Fig. 5G, Supplementary Table S4). Potato chipping was performed using tubers stored under 22 °C and 4 °C, respectively. Potato chips processed from tubers stored under 22 °C showed a similar color from all 3 lines as well as WT ‘Katahdin’ (Supplementary Fig. S7). After the 4 wks of storage of the tubers under 4 °C, chips from KV78, KV87, and KV108 all showed a lighter color than those from ‘Katahdin’ (Supplementary Fig. S7).

Collectively, these results showed that although the deletions associated with *VInv*In2En of ‘Katahdin’ are in heterozygous and mosaic conditions in the 3 T0 CRISPR/Cas lines, the deletions resulted in a significant reduction of *VInv* expression under cold storage condition, confirming the cold-responsive function of *VInv*In2En in ‘Katahdin.’

### Evolution of *VInv* gene and *VInv*In2En enhancer

**Figure 6.**
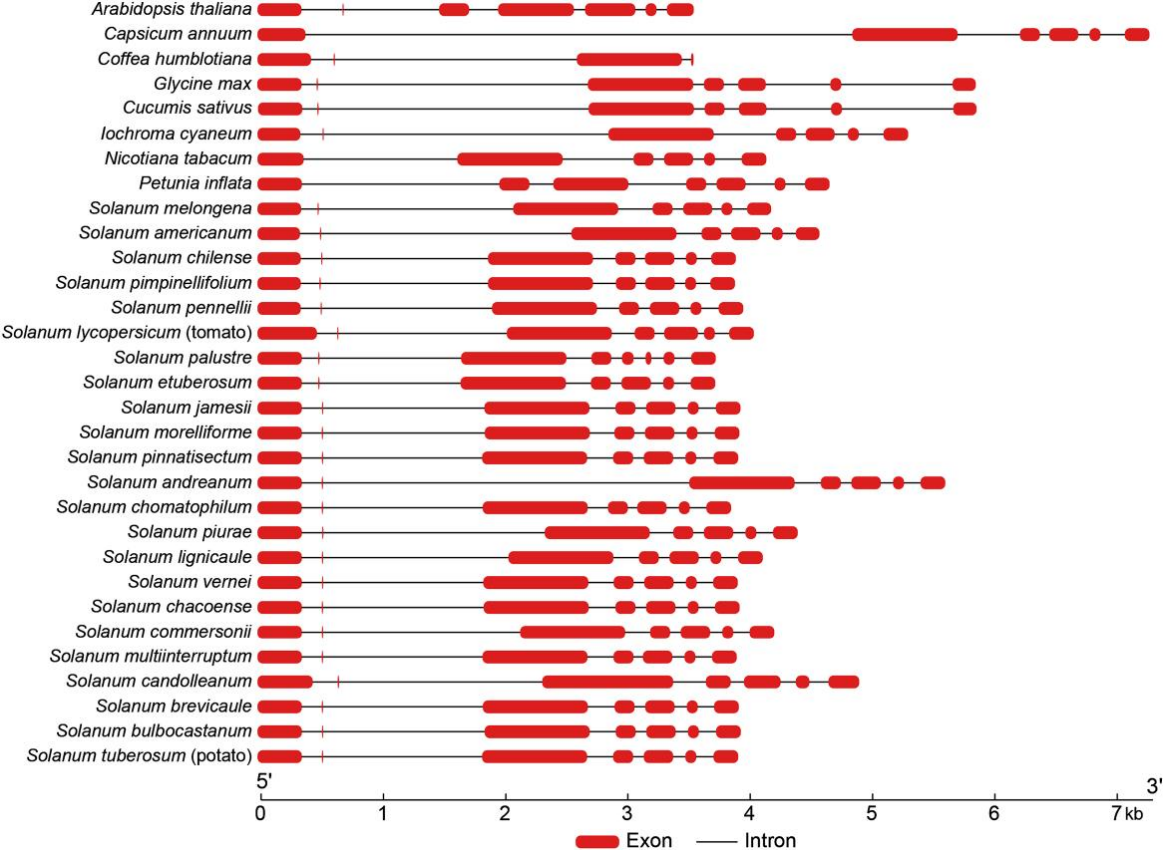
Composition of introns and exons of *VInv* genes from different plant species. A total of 28 Solanaceous species and 8 distantly related dicot species were selected for the analysis. The distinctly small exon 2 (9 bp) was detected in all Solanaceous species, as well as in 5 distantly related plant species. In addition, a large intron 2 (ranging from 780 to 2,997 bp) following the small exon 2 was identified in most plant species.

We computationally extracted the DNA sequence of *VInv* gene from a total of 28 sequenced Solanaceous species. Sequences from several distantly related species, including *A. thaliana*, cucumber (*Cucumis sativus*), and soybean (*Glycine max*), were used as outgroups in evolutionary analysis. The VINV protein of potato shared 92% to 99% sequence similarity with those from tomato and wild *Solanum* species (Supplementary Fig. S8). In addition, the structure of the *VInv* genes is also highly conserved among different species (Fig. 6). The distinct small exon 2 (9 bp) was detected in all Solanaceous species, as well as in several distantly related plant species. In addition, a large intron 2 was identified following the small exon 2 in all species (Fig. 6), with sizes ranging from 780 bp to 2,997 bp (Supplementary Table S5).

The 1,327 bp intron 2 sequence from RH (Zhou *et al*. 2020) was used to align intron 2 sequences from other Solanaceous species. Homologous sequences were detected in the same intron of the *VInv* gene from all *Solanum* species, as well as from several distantly related species, including eggplant (*Solanum melongena*) and pepper (*Capsicum annuum*) (Supplementary Table S5). We next aligned the 200 bp *VInv*In2En sequence from RH to intron 2 sequences from different species. Surprisingly, the *VInv*In2En sequences were more conserved than intron 2 sequences among the species analyzed (Supplementary Table S5). Furthermore, the DNA motifs related to CBF/NF-Y, TCP, and GATA were detected in the *VInv*In2En sequences from distantly related *Solanum* species (Supplementary Table S5). Therefore, *VInv*In2En represents a conserved enhancer sequence in *Solanum* species.

We extracted the *VInv*In2En sequence from several different potato genotypes to further exploit its sequence polymorphism (Supplementary Fig. S9), including diploid potato clones M6 (Jansky *et al*. 2014) and H28-7 (Bhaskar *et al*. 2010), which are resistant to CIS. SNPs and small indels were observed throughout the *VInv*In2En sequence in comparison between CIS-resistant (H28-7 and M6) and CIS-susceptible (RH) genotypes, including SNPs located in the CBF/NF-Y, GATA, and TCP motifs (Supplementary Fig. S9). Thus, sequence polymorphism of *VInv*In2En may contribute to the level of CIS resistance of different potato genotypes.

## Discussion

Invertases hydrolyze sucrose into glucose and fructose, thereby playing important roles in metabolism and development in plants (Ruan *et al*. 2010). Different plant invertases have been found to be specific to the cell wall, vacuole, or cytosol, respectively. Both cell wall and vacuolar invertases are also known to contribute to defense responses to abiotic and biotic stresses (Wan *et al*. 2018). Vacuolar invertases play essential roles in cell expansion and sugar accumulation, which are related to plant growth and development (Ruan *et al*. 2010; Wan *et al*. 2018). Therefore, silencing of the vacuolar invertase gene can cause major developmental defects in plants. For example, silencing of the vacuolar invertase gene in tomato (*Solanum lycopersicum*) resulted in substantially smaller fruits (Klann *et al*. 1996). Major developmental defects were also reported in silencing of the vacuolar invertase gene in several other species, including carrot (*Daucus carota*) (Tang *et al*. 1999), muskmelon (*Cucumis melo*) (Yu *et al*. 2008), cotton (*Gossypium hirsutum*) (Wang *et al*. 2014; Wang and Ruan 2016), and rice (*Oryza sativa*) (Lee *et al*. 2019; Deng *et al*. 2020).

*VInv* (*Pain-1*) is the only vacuolar invertase gene identified in the potato genome (Bhaskar *et al*. 2010; Draffehn *et al*. 2010). Interestingly, silencing of the *VInv* gene by RNAi in potato did not cause unambiguous defects in growth and development (Bhaskar *et al*. 2010). The potato RNAi lines did not show yield loss in field-based yield trials (Bhaskar *et al*. 2010). These results suggest that the *VInv* gene may not play a similar developmental role in potato as compared with other plant species. Although *VInv* is expressed in nontuber tissues, the expression of *VInv* is not upregulated by cold stress in several nontuber tissues, including petiole, stem, and root (X.B. Zhu, unpublished data). Similarly, the GUS signals in the transgenic *A. thaliana* plants derived from *VInv*In2 and *VInv*In2En constructs were not enhanced by cold stress. We hypothesize that the *VInv* gene has adapted for a distinct role in the tuber-bearing species in response to cold stress. A high level of *VInv* expression at cold temperatures would generate more sugars in tuber cells, which in turn would affect the osmotic pressure and increase the freezing tolerance of tuber cells that contain a high percentage of water.

The *VInv*In2En sequence is conserved among distantly related *Solanum* species, including tomato and several other non-tuber-bearing species (Supplementary Table S5 and Fig. S9). Thus, *VInv*In2En emerged before the divergence between tuber-bearing and non-tuber-bearing species. We speculate that *VInv*In2En contains unidentified sequence motif(s) that are responsible for its tuber-specific function. We previously showed that the CIS-resistant diploid potato germplasm line H28-7 exhibits a very low level of *VInv* expression in cold-stored tubers (Bhaskar *et al*. 2010). Interestingly, we detected a SNP in each of the 2 CBF/NF-Y motifs in *VInv*In2E between H28-7 and RH (Supplementary Fig. S9). These results suggest that variation of the *VInv*In2En sequence is likely the key factor for the resistance of the CIS-resistant germplasm. In contrast, an identical *VInv*In2En sequence was observed in DM1-3 and RH potatoes (Supplementary Fig. S9), which have different levels of resistance to CIS. Thus, the *VInv*-mediated cold tolerance is likely associated with additional factors depending on species or genotypes within a species. This hypothesis is supported by previous reports demonstrating an invertase inhibitor, StInvInh2, which specifically suppresses the activity of the VINV protein (Liu *et al*. 2010; Brummell *et al*. 2011). A combination of *VInv*In2En-mediated cold-induced expression of *VInv* and posttranscriptional regulation of VINV protein provides a multilayer of defense system for potato to adapt to different environments and/or stress conditions.

Several TFs, including CBF/NF-Y, TCP, and GATA, may play a role in *VInv*In2En-mediated regulation of *VInv* under cold conditions, since mutations of the predicted binding sites of these TFs abolished the function of *VInv*In2En as a transcriptional enhancer in *A. thaliana* (Fig. 3). CBF/NF-Y, TCP, and GATA are large TF families in plants and include 41, 31, and 49 genes, respectively, in the potato genome (Wang *et al*. 2019; Li *et al*. 2021; Yu *et al*. 2022). Although there are no reports yet on cold response associated with these TFs in potato, specific members from the CBF/NF-Y, TCP, and GATA families have been documented for playing a role in cold temperature response in other plant species. For example, a GATA-family TF in rice, OsGATA16, was induced by cold treatment and can improve cold tolerance by repressing some cold-related genes (Zhang *et al*. 2021). A TCP1 TF in *Chrysanthemum morifolium*, DgTCP1, was induced by cold temperature and can regulate peroxidase activity and reduce ROS accumulation (Li *et al*. 2022a). It is interesting to note the presence of 3 CBF/NF-Y binding sites in close vicinity within *VInv*In2En. The NF-Y TFs have been documented to confer response to various types of abiotic stresses, including drought, salt, nutrient, and temperature (Zhang *et al*. 2023). Thus, it will be essential to validate the functions of these TF-binding sites in potato and to identify a specific member(s) from these TF families that are responsible for the function of *VInv*In2En.

## Materials and methods

### Enhancer validation using transgenic assays in potato

An intronic DHS within intron 2 of *VInv* gene was identified from the DHS data published previously (Zeng *et al*. 2019). The entire intron 2 from the *VInv* gene of RH potato (*S. tuberosum*) was used for enhancer validation using a GUS reporter system (Zhu *et al*. 2015). The forward (*VInv*In2) and reverse (*VInv*In2R) sequences of intron 2 were amplified from genomic DNA of RH potato using PCR with primers VIT-F6/R6 and VIT-F8/R8 (Supplementary Table S3), respectively, and were ligated to a minimal 35S promoter (−50 to −2 bp) (m35S) through the *Eco*RI cloning site. The ligated PCR products were cloned into the pENTR/D directional TOPO cloning vector (Invitrogen) and then transferred into the pKGWFS 7.0 vector containing the GUS reporter using the LR Clonase recombination method (Zhu *et al*. 2015). Constructs were transferred into *Agrobacterium tumefaciens* strain GV3101 (pMP90), followed by transformation to potato variety ‘Katahdin’ using methods described previously (Bhaskar et al. 2008; Bhaskar et al. 2010).

Transgenic ‘Katahdin’ lines derived from the forward or reverse construct were obtained and screened using PCR with the kanamycin gene-specific primers Kan-F/R and the construct-specific primers (Supplementary Table S3). All transgenic lines with 3 replicates for each line were grown in greenhouses using photoperiod of 16 h daylight at 22 °C and 8 h darkness at 16 °C (50% to 70% humidity) and light intensity of 500 *μ*mol/m^2^/s (natural light combined with light of high-pressure sodium lamps) until leaves became senesced naturally. Tubers harvested from each line were divided into 2 groups: stored in dark at 22 °C (50% to 70% humidity) or 4 °C (60% to 70% humidity) for 4 wks, respectively. Tuber slices prepared by slicing longitudinal sections 2 mm thick from the center of individual tubers were examined for GUS activity. Tuber slices were placed in a plastic plate (70 × 15 mm) and soaked in GUS staining solution (100 mm sodium phosphate, pH 7.0, 10 mm EDTA, 0.1% [*v*/*v*] Triton X-100, 0.5 mm potassium ferrocyanide, 0.5 mm potassium ferricyanide, and 0.05% [w/v] X-Gluc), with vacuum infiltration for 30 min and incubation in dark at 37 °C overnight. Tuber slices were washed in 80% (*v*/*v*) ethanol several times. Images of tuber slices were captured using an EPSON Perfection 4180 scanner.

### Enhancer dissection using transgenic assays in *A. thaliana*

Seeds of Arabidopsis (*A. thaliana*) accession Col-0 were germinated in half-strength MS (0.5× MS) medium, and the seedlings were transplanted in potting soil and grown in plant growth chambers with 16/8 h light/dark cycles at 23 °C and light intensity of 150 *μ*mol/m^2^/s (white fluorescent lamps) until flowering. The *VInv*In2 and *VInv*In2R constructs were initially used to transform *A. thaliana* accession Col-0 using the floral dip method (Clough and Bent 1998). Transgenic seedlings were screened on solid 0.5× MS medium containing kanamycin (50 *μ*g/mL) and were grown in an illumination incubator with the same light–dark condition described above and were examined for GUS activity according to published protocols (Zhu *et al*. 2015).

To map the position of the enhancer within intron 2 of *VInv*, we divided intron 2 into 10 DNA fragments (#1 to #10) using 5 breaks (b1 to b5) for transgenic assays. The stem/petiole-specific enhancer (within DNA fragment #11) was further divided into 14 (#11 to #24) subfragments. All target DNA fragments together with the m35S were synthesized from GenScript Inc. and cloned into the pKGWFS 7.0 vector containing the m35S and the *GUS* reporter gene (Zhu *et al*. 2015). Images of transgenic *A. thaliana* seedlings were captured using the EPSON Perfection 4180 scanner to record the GUS signals.

### Analysis of TF-binding motifs

TF-binding motifs and their corresponding TFs within intron 2 of *VInv* were identified using 2 independent programs of CIS-BP (Weirauch *et al*. 2014) and PlantPAN 3.0 (Chow *et al*. 2019) with default parameters. DNA motifs consistently detected by both programs were used for further analysis. Motifs reported to be associated with cold response in 1 or multiple plant species were mapped to intron 2 of *VInv* using TBtools (Chen *et al*. 2020).

### Development of CRISPR/Cas deletion lines

A self-compatible diploid potato clone DMF5-73-1 was developed from a cross between *S. tuberosum* Gp. Phureja DM 1-3 516 R44 (DM1-3) and *Solanum chacoense* (M6) (Endelman and Jansky 2016) and has been self-pollinated for 5 generations. WT and CRISPR/Cas lines were propagated in vitro on MS medium (MS basal salts plus vitamins, 3% sucrose, 0.7% plant agar, pH 5.8) (Murashige and Skoog 1962). In vitro plants were maintained in growth chambers with 16 h light/8 h dark photoperiod at 22 °C and average light intensity of 200 *μ*mol/m^2^/s (white fluorescent lamps) *Pro*.

The Csy4-based CRISPR/Cas9 system (Cermak *et al*. 2017) was used to develop *VInv*In2En deletion lines in DMF5-73-1. In brief, 5 sgRNAs flanking *VInv*In2En (1a, 2a, 3a, 1b, and 2b) and a single sgRNA (3b) targeting *VInv*In2En (Supplementary Table S2) were designed using program of CRISPR-P v2.0 (Liu *et al*. 2017). The 6 gRNAs were linked by Csy4 binding sites and then cloned into the Csy4 multiplexing vector (Supplementary Fig. S3) based on published methods (Cermak *et al*. 2017). The construct was delivered into *A. tumefaciens* GV3101 (pMP90) and was used to conduct hairy root-based *Agrobacterium* transformation (Butler *et al*. 2020). T0 CRISPR/Cas lines showing the expected smaller PCR products were further confirmed by Sanger sequencing using *VInv*-mut-F1/R1 primers (Supplementary Table S3). Several T0 lines with large deletion of *VInv*In2En were grown under greenhouse conditions as described above, followed by subsequent self-pollination to obtain homozygous T1 deletion lines.

A tetraploid potato cultivar ‘Katahdin’ was used to develop deletion lines using the U3/U6-based CRISPR/Cas9 system (Hu *et al*. 2019). Four sgRNAs, including R1 outside of *VInv*In2En and R2, R3, and R4 inside the *VInv*In2En (Supplementary Table S2), were designed using CRISPR-P v2.0 (Liu *et al*. 2017). The sgRNAs were assembled into 4 expression cassettes (*ProAtU3b:gRNA1*, *ProAtU3d:gRNA2*, *ProAtU6-29:gRNA3*, and *ProAtU6-29:gRNA4*), which were cloned into the pHNCas9 vector by using the Golden Gate cloning strategy (Ma et al. 2015; Xie et al. 2015; Ma et al. 2016; Hu et al. 2019). The construct pHNCas9::*VInv*In2En was introduced into *A. tumefaciens* GV3101 (pMP90) and was used to transform ‘Katahdin’ according to published protocols (Bhaskar *et al*. 2008). Positive transformants were screened using PCR with primers *Kan*-F3/R3, *Cas*-F1/R1, and *VInv*-Edit-F/R (Supplementary Table S3). Transgenic lines containing additional smaller bands (2% agarose gel) were further confirmed by Sanger sequencing. PCR products were purified by using QIAquick PCR Purification Kit (Qiagen) and were cloned into *Escherichia coli* using pMD 19-T vector (TaKaRa). A minimum of 60 randomly selected positive colonies derived from each deletion line were fully sequenced. Statistical analysis of different types of deletions was conducted on each of the ‘Katahdin’ CRISPR/Cas deletion lines containing 3 haplotypes, A (2 copies), B, and C.

### Greenhouse trials, tuber sample preparation, and chipping analysis

Each of 10 seed tubers of RH potato was planted in potting soil under normal greenhouse conditions as described above. Standard cultivation and management practices were followed throughout the growing period. Tubers were harvested 120 d after seedling emergence when leaves senesced naturally. Tubers harvested from 2 pots were combined together as 1 biological replicate. Tubers of 5 biological replicates were stored in dark at 22 °C (50% to 70% humidity) for 10 d and then divided into 2 groups. Each group was stored in dark at 22 °C (50% to 70% humidity) or 4 °C (60% to 70% humidity) for 0, 2, 4, 8, and 16 wks, respectively.

Three T0 CRISPR/Cas deletion lines (3 plants for each line) developed from ‘Katahdin’ were grown under normal greenhouse conditions as described above. Tubers harvested from the same line were combined together and stored under dark at 22 °C for 10 d and then divided into 2 groups for 22 °C (50% to 70% humidity) or 4 °C (60% to 70% humidity) treatments, and each group of tubers with 3 replicates was treated for 2 and 4 wks, respectively.

Tuber samples of 1.5-mm-thick slices (1 to 3 slices for each tuber) prepared from the apical to basal end of the tuber were taken for chipping analysis. The remaining tuber samples were frozen in liquid nitrogen and used for analysis of *VInv* expression. Tuber slices were fried in cottonseed oil at 191 °C for 2 min or until the cessation of bubbles. Chip color of cold-stored tubers is compared with that of the corresponding controls.

### *VInv* transcription and splicing assays

RNAs were extracted from tuber tissues using Plant RNA Isolation Mini Kit (Agilent) following the manufacturer's instructions and were reverse transcribed to cDNAs using Invitrogen SuperScript III Reverse Transcriptase Kit (Invitrogen) with oligo(dT)_20_ primer. *VInv* transcripts were quantified by RT-qPCR using the SYBR Advantage qPCR Premix (Clontech) with the specific primers for *VInv* and the reference gene *Actin97* described previously (Zhu et al. 2014; Zhu et al. 2016). RT-qPCR was performed on the CFX96 Touch Real-Time PCR Detection System (Bio-Rad) with a program of 30 s at 95 °C, 40 cycles of 10 s at 95 °C, 20 s at 60 °C for *VInv* and *Actin97*, and 30 s at 72 °C, followed by a plate read. Next is 2 s at 50 °C to 95 °C with 0.2 °C steps for melting curve, followed by a final extension step of 10 min at 72 °C. Relative expression levels of *VInv* gene were calculated using Gene Expression Macro software version 1.1 (Bio-Rad Laboratories). Data for each treatment are presented as Se of the means of the 3 biological replicates. For RT-qPCR data of 3 ‘Katahdin’ CRISPR/Cas9 lines and the WT, ANOVA was carried out using PROC GLM in the Statistical Analysis System version 9.1 (SAS v9.1) (SAS Institute Inc., Cary, NC) (Supplementary Table S4).

To examine whether *VInv*In2En deletions affect *VInv* gene splicing, we prepared cDNAs from tuber tissues of the 3 ‘Katahdin’ CRISPR/Cas lines. Exon 1 to exon 3 of *VInv* was amplified using primers Splicing-F/R (Supplementary Table S3; amplicon size: 612 bp). The RT-PCR products were purified by using QIAquick PCR Purification Kit (Qiagen) and then used for Sanger sequencing.

### Y1H assay

Triple copies of the *VInv*In2En sequence (*VInv*In2En*3) were synthesized and used to develop a bait plasmid p*VInv*In2En*3-AbAi. The bait plasmid was used to screen the cDNA library, developed from cold-treated tuber tissues of RH potato, according to methods described in the Matchmaker Gold Yeast One-Hybrid Library Screening System User Manual (Clontech, http://www.takarabio.com/). Yeast (*Saccharomyces cerevisiae*) colonies were cultured on plates containing SD/-Leu/AbA^200 ng/mL^ medium at 30 °C for 3 to 5 d, and those >2 mm in diameter were analyzed by PCR amplification and Sanger sequencing using primers pGADT7-F/R (Supplementary Table S3). The resulted sequences from the 387 yeast colonies were used for BLAST search in the DM1-3 potato cDNA (v6.1) database by using Spud DB BLASTn program with default parameters (http://spuddb.uga.edu/blast.shtml). We identified the best matched cDNA sequence for each of the 387 sequences. The cDNA sequences with an *E*-value < 1e^−5^ and a minimum sequence identity of 82% were kept for further analyses. We identified a total of 33 unique cDNA sequences after filtering out repetitive cDNA sequences.

To further validate the interactions between the candidate proteins and the *VInv*In2En enhancer, point-to-point Y1H assay was performed. Full-length CDSs of the candidate proteins related to the 33 identified cDNAs were inserted into the prey vector pGADT7 by using HB-infusion Cloning Kit (HANBIO, https://www.hanbio.net/en/company.shtml/). The prey plasmids were subsequently transformed into the bait yeast strain Y1HGold[p*VInv*In2En*3-AbAi] by using the Yeastmaker Yeast Transformation System 2 (Clontech, http://www.takarabio.com/). The yeast colonies were transferred to plates containing SD/-Leu/AbA^200 ng/mL^ medium and then allowed to grow at 30 °C for 3 d.

### Analysis of *VInv* evolution

A total of 28 Solanaceous species (https://solgenomics.net/) (Tang *et al*. 2022) and several other dicot species (Supplementary Table S5), including *A. thaliana*, cucumber (*C. sativus*), and soybean (*G. max*), were selected for evolutionary analysis of the *VInv* gene. Information on evolutionary timescale of life for all 31 species were collected from the TimeTree 5 database (http://www.timetree.org/) (Kumar *et al*. 2022) and visualized in MEGA X software (Kumar *et al*. 2018).

Protein sequences of *VInv* gene from the 31 different plant species were extracted and aligned to that from RH potato using the National Center for Biotechnology Information (NCBI) BLASTp program (https://blast.ncbi.nlm.nih.gov/Blast.cgi). The intron and exon composition of the *VInv* gene from 31 plant species was analyzed using the online tool GSDS 2.0 (Hu *et al*. 2015). The 1,327 bp intron 2 and the 200 bp *VInv*In2En sequences from RH were used to align intron 2 sequences from other 30 species to identify homologous sequences using the program of NCBI BLASTn.

### Accession numbers

Sequence data from this article can be found in the GenBank/EMBL libraries under the following accession numbers: Soltu.DM.03G015280 (*VInv*), Soltu.DM.01G024340 (StNF-YC9), and Soltu.DM.06G027230 (StNF-YC1). All mutants and transgenic lines are described in Supplementary Table S6.

## Supplementary Material

koae050_Supplementary_Data

## Data Availability

All materials, including constructs, are available upon request.

## Supplementary data

The following materials are available in the online version of this article.

**Supplementary Figure S1.** *VInv* expression and its impact on chip color of RH potato.

**Supplementary Figure S2.** Binary vector for delivering CRISPR/Cas9 components targeting *VInv*In2En in diploid potato DMF5-73-1.

**Supplementary Figure S3.** Genotyping of homozygous CRISPR/Cas deletion lines developed from DMF5-73-1.

**Supplementary Figure S4.** Three haplotypes associated with the middle portion of intron 2 of *VInv* gene from tetraploid potato ‘Katahdin.’

**Supplementary Figure S5.** Binary vector for delivering CRISPR/Cas9 components targeting *VInv*In2En in ‘Katahdin.’

**Supplementary Figure S6.** Deletions within *VInv*In2En of ‘Katahdin.’

**Supplementary Figure S7.** Potato chips processed from the 3 CRISPR/Cas9 deletion lines and WT ‘Katahdin.’

**Supplementary Figure S8.** Evolution of the VINV protein and enhancer *VInv*In2En.

**Supplementary Figure S9.** Alignment of the *VInv*In2En sequence from tomato and 4 different diploid potato genotypes.

**Supplementary Table S1.** Sequences and mutated nucleotides within the 200 bp *VInv*In2En.

**Supplementary Table S2.** List of sgRNAs used in all CRISPR/Cas experiments.

**Supplementary Table S3.** List of primers for GUS reporter, CRISPR/Cas-based genome editing, splicing, and Y1H.

**Supplementary Table S4.** ANOVA for RT-qPCR data from the ‘Katahdin’ CRISPR/Cas9 lines.

**Supplementary Table S5.** Sequence analysis of intron 2 and enhancer VInvIn2En from different plant species.

**Supplementary Table S6.** Mutants and transgenic lines.

## Dive Curated Terms

The following phenotypic, genotypic, and functional terms are of significance to the work described in this paper:

DHS Gramene: AT5G05920
DHS Araport: AT5G05920

TCP1 Gramene: AT1G67260
TCP1 Araport: AT1G67260
