# Supplementary material for: Molecular dissection of an intronic enhancer governing cold-induced expression of the vacuolar invertase gene in potato
Source: Plant Cell. 2024 Feb 20;36(5):1985–99. doi: 10.1093/plcell/koae050 (PMC11062429; doi:10.1093/plcell/koae050)
Supplement: koae050_Supplementary_Data [file koae050_supplementary_data.zip › koae050_Supplementary_Data.pdf]

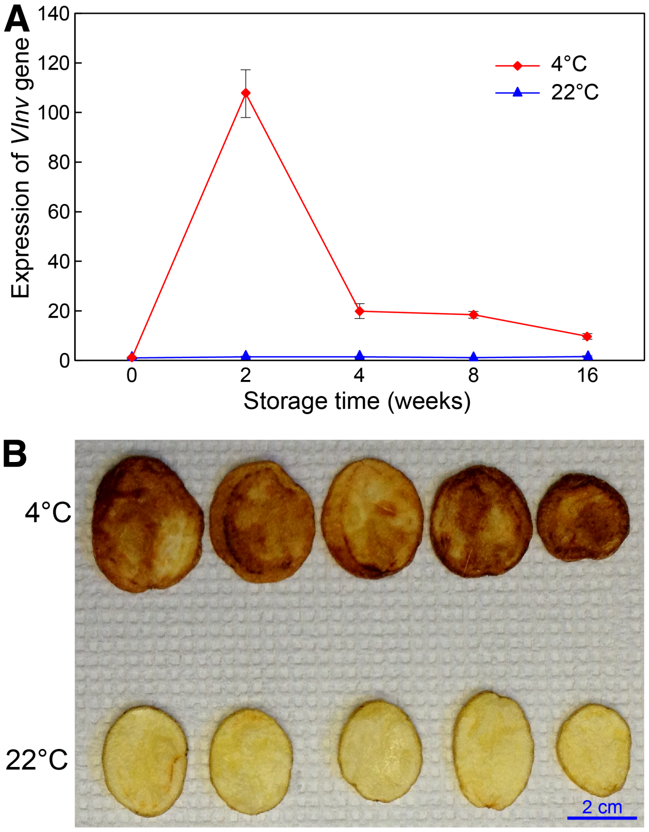

Supplemental Figure S1. *VInv* expression and its impact on chip color of RH potato. (Supports Figure 1)

(**A**) Reverse transcription quantitative PCR-based transcription profiles of the *VInv* gene in potato tubers after 0, 2, 4, 8, 16 weeks of storage under 22°C and 4°C, respectively. *VInv* expression is normalized relative to the potato reference gene *Actin97*. The relative expression level of *VInv* at 0 week was defined to 1. Each data point represents mean ± standard error from three biological replicates.

(**B**) Potato chips were processed from RH tubers harvested from greenhouse-grown plants and stored at room temperature (22°C) and cold temperature (4°C) for 4 weeks, respectively. The scale bar represent 2 cm.

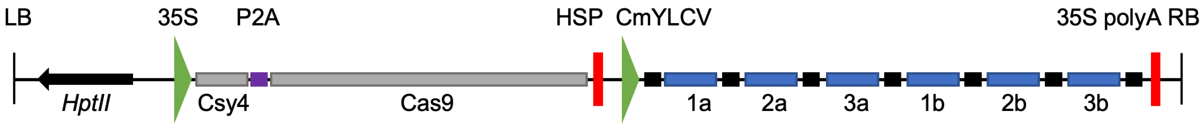

Supplemental Figure S2. Binary vector for delivering CRISPR/Cas9 components targeting *VInv*In2En in diploid potato DMF5-73-1. (Supports Figure 5)
The hygromycin resistance marker (*HptII*, black arrow) was used for plant selection and 35S and CmYLCV promoters (green arrows) were used for expressing enzymes, Csy4 and Cas9 (gray boxes) and sgRNAs 1a, 2a, 3a, 1b, 2b, and 3b (blue boxes). Csy4 binding sites (black boxes) separate sgRNAs and were used for sgRNA processing. LB and RB represent the left border and right border, respectively.

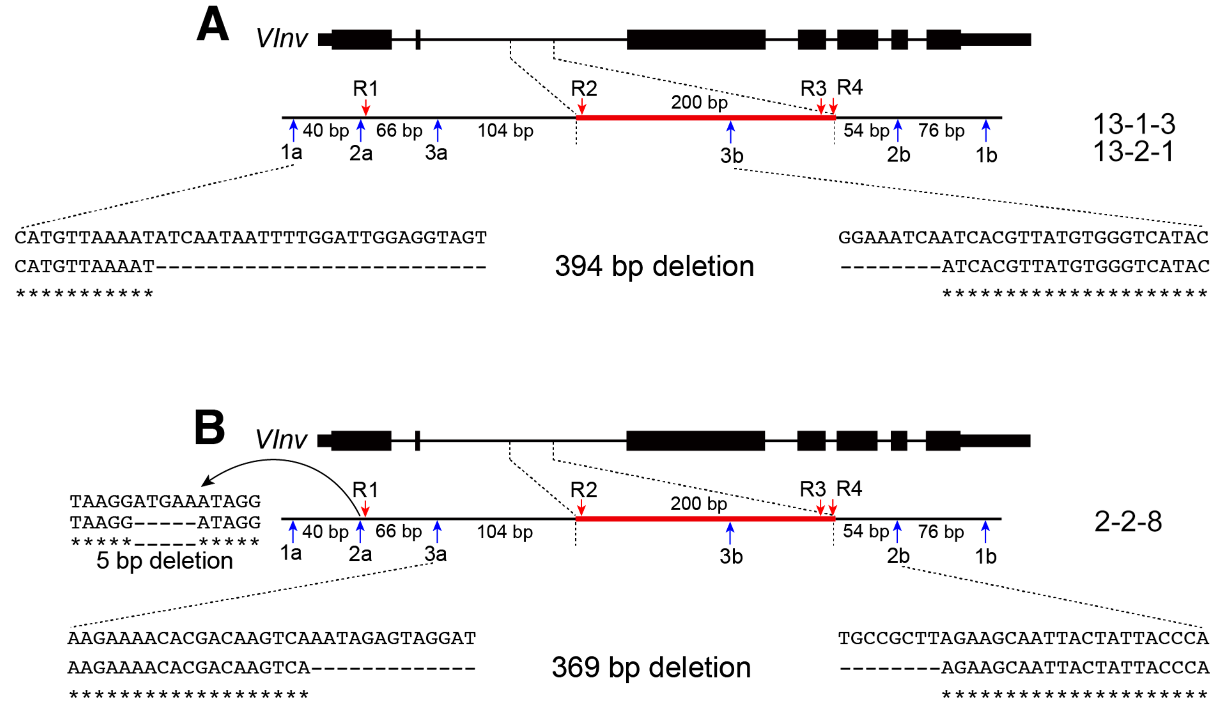

Supplemental Figure S3. Genotyping of homozygous CRISPR/Cas deletion lines developed from DMF5-73-1. (Supports Figure 5)
Red bars mark the 200-bp *VInv*In2En enhancer. Red arrows indicate the positions of the four sgRNAs (R1, R2, R3, R4) within and outside of *VInv*In2En. Blur arrows indicate the positions of the six sgRNAs (1a, 2a, 3a, 1b, 2b, 3b) within and outside of *VInv*In2En.
(**A**) A 394-bp homozygous deletion spanning sgRNA 1a and 3b was detected in lines 13-1-3 and 13-2-1. Note: T0 events for both 13-1-3 and 13-2-1 were regenerated from the same primary transformant hairy root culture.
(**B**) A 369-bp homozygous deletion spanning sgRNA 3a and 2b and a 5-bp deletion within sgRNA 2a were detected in line 2-2-8. All three homozygous lines were generated from DMF5-73-1 background by selfing a T0 event transformed with the CRISPR/Cas binary vector (Supplemental Figure S2). Genotyping was conducted by PCR using primers *VInv*-mut-F1/R1 and Sanger sequencing.

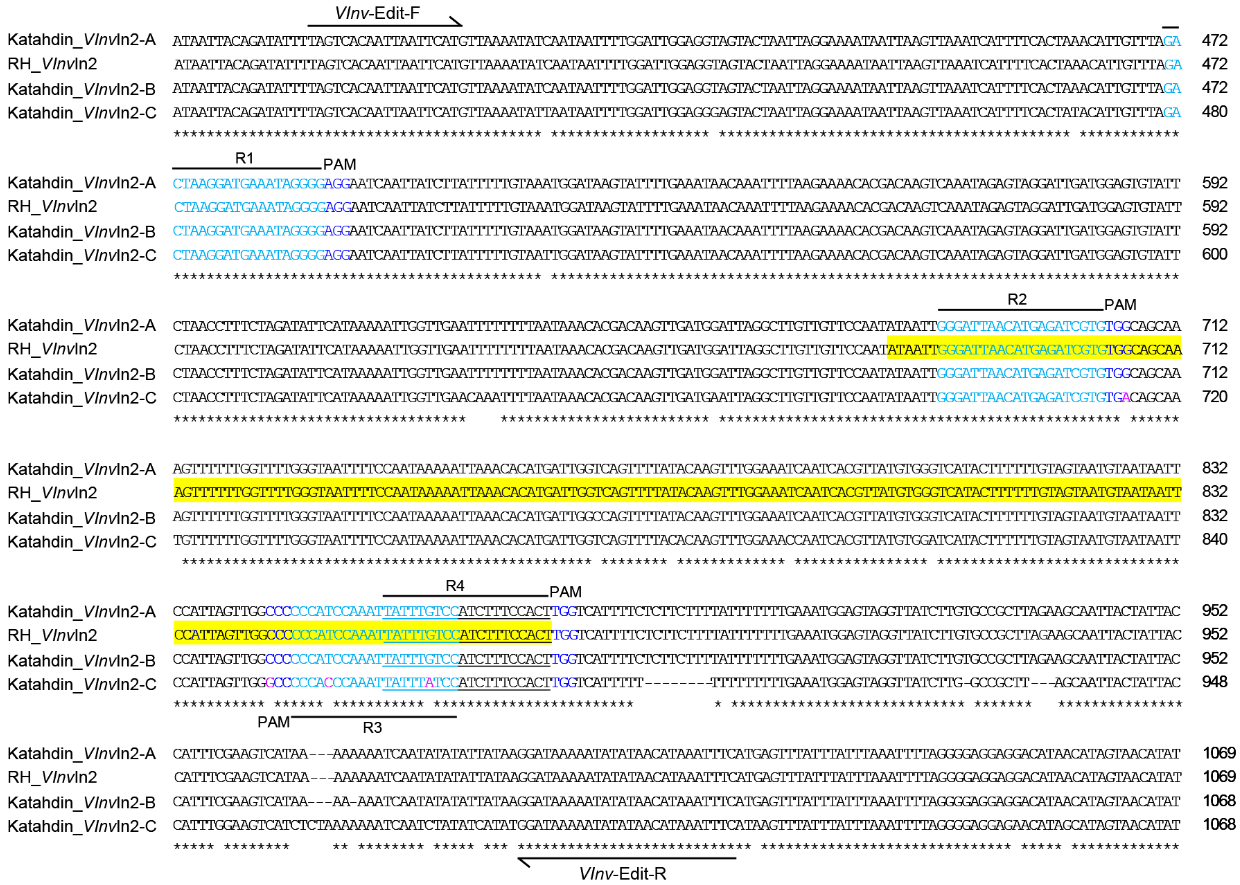

Supplemental Figure S4. Three haplotypes associated with the middle portion of intron 2 of *VInv* gene from tetraploid potato Katahdin. (Supports Figure 5)
The 200-bp *VInv*In2En from RH potato is highlighted in yellow. The positions of the four sgRNAs (R1, R2, R3 and R4) are marked in turquoise. The protospacer adjacent motif (PAM) sequences are highlighted in blue. “*” indicates identical nucleotides conserved among all haplotypes. Nucleotides highlighted in purple are associated single nucleotide polymorphisms (SNPs) between haplotype A and haplotype C. Sequences indicated by arrows are target sites of PCR primers VInv-Edit-F and VInv-Edit-R, respectively.

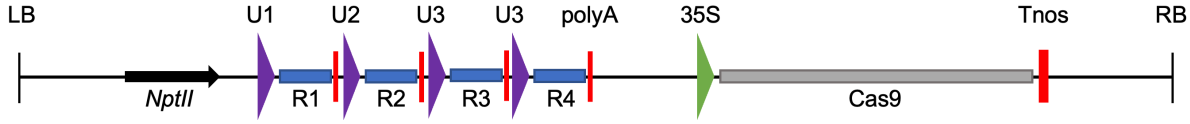

Supplemental Figure S5. Binary vector for delivering CRISPR/Cas9 components targeting *VInv*In2En in Katahdin. (Supports Figure 5)
The Kanamycin resistance marker (*NptII*, black arrow) was used for plant selection and 35S promoter (green arrow) was used for expressing Cas9 enzyme (gray box), and promoters U1 (U3b), U2 (U3d), U3 (U6-29), and U3 (U6-29) (purple arrows) were used for expressing sgRNAs R1, R2, R3, and R4 (blue boxes). Red bars indicate where the expression is terminated. LB and RB represent the left border and right border, respectively.

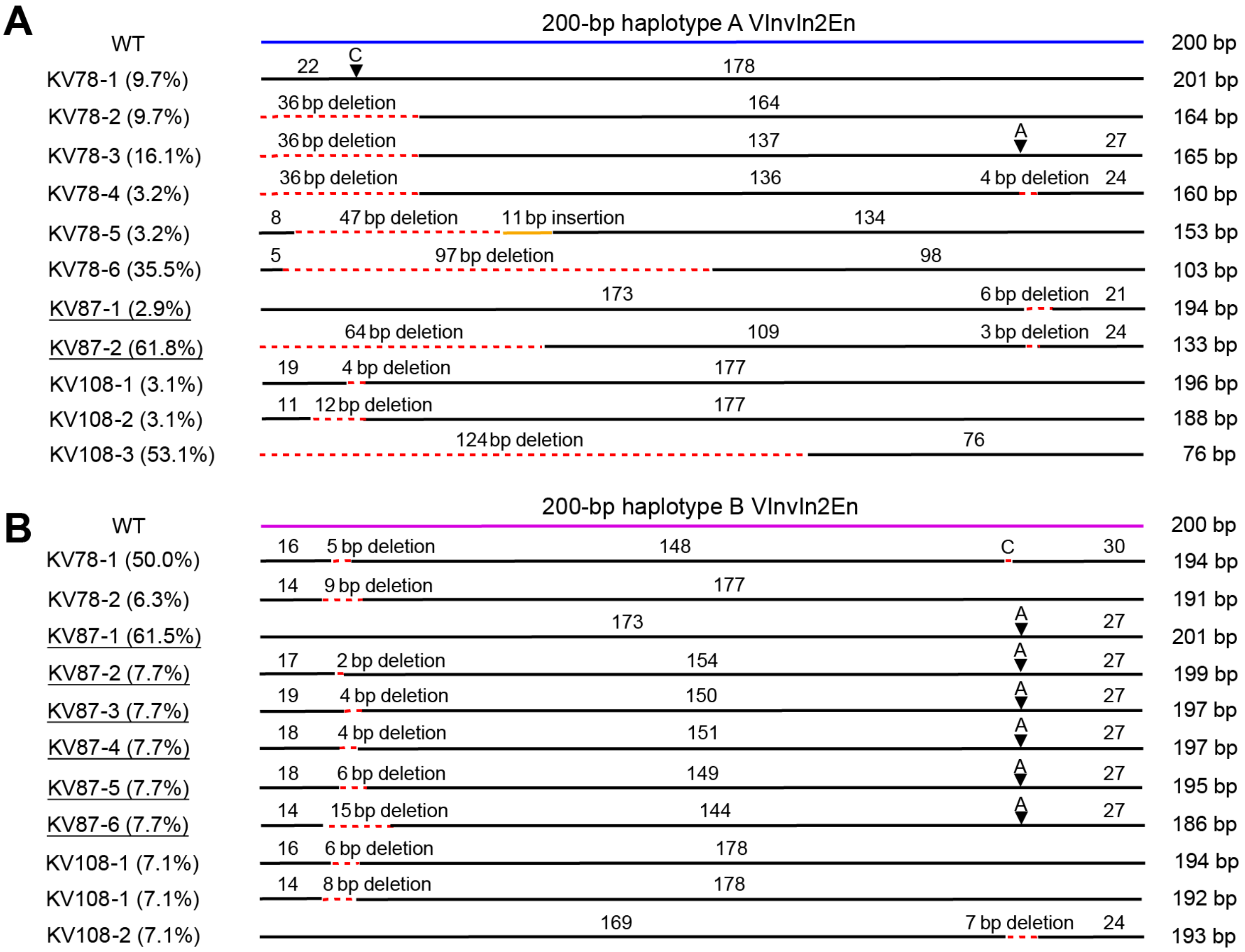

Supplemental Figure S6. Deletions within *VInv*In2En of Katahdin. (Supports Figure 5)
(**A**) Different deletions associated with haplotype A of *VInv*In2En.
(**B**) Different deletions associated with haplotype B of *VInv*In2En.
Sequence variants were detected in three T0 CRISPR/Cas9 lines (KV78, KV87 and KV108). Red dot or dotted lines represent deletions in different sizes. Insertions of single nucleotide are pointed by arrowheads. Percentages in brackets indicate the percentage of sequences with deletion/mutation in total number of sequences, including the wild type of haplotype A *VInv*In2En or haplotype B *VInv*In2En. The actual fragment lengths for different type of deletions are listed on the right. All numbers above black lines indicate base pairs. The wild type (WT) *VInv*In2En are marked in blue (haplotype A) and purple (haplotype B), respectively.

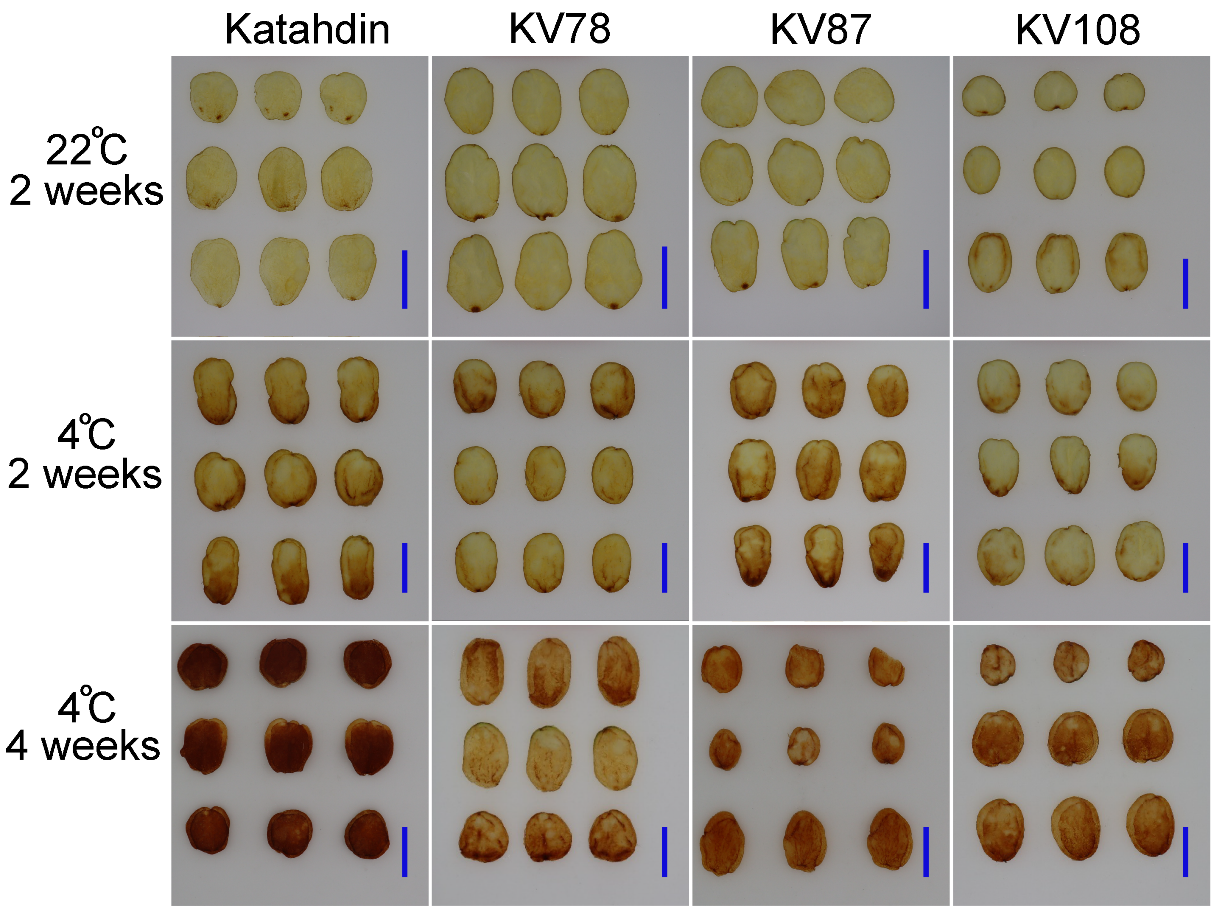

Supplemental Figure S7. Potato chips processed from the three deletion lines and wild type Katahdin. (Supports Figure 5)
Tubers were stored at 22°C for 2 weeks, or at 4°C for 2 weeks and 4 weeks, respectively. The vertical blue bars represent 5 cm.

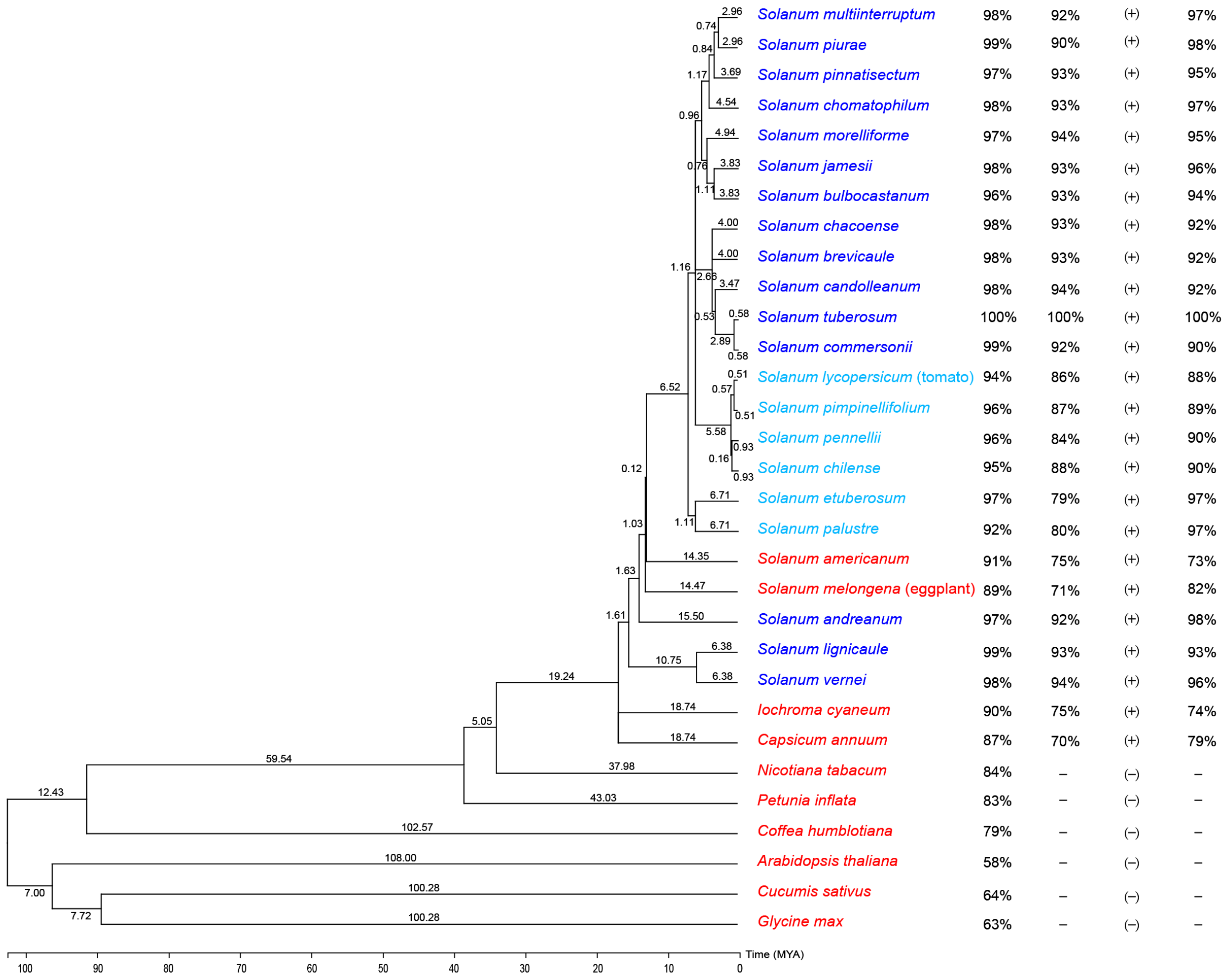

Species	A	B	C	D
Solanum multiinterruptum	98%	92%	(+)	97%
Solanum piurae	99%	90%	(+)	98%
Solanum pinnatisectum	97%	93%	(+)	95%
Solanum chomatophilum	98%	93%	(+)	97%
Solanum morelliforme	97%	94%	(+)	95%
Solanum jamesii	98%	93%	(+)	96%
Solanum bulbocastanum	96%	93%	(+)	94%
Solanum chacoense	98%	93%	(+)	92%
Solanum brevicaule	98%	93%	(+)	92%
Solanum candolleanum	98%	94%	(+)	92%
Solanum tuberosum	100%	100%	(+)	100%
Solanum commersonii	99%	92%	(+)	90%
Solanum lycopersicum (tomato)	94%	86%	(+)	88%
Solanum pimpinellifolium	96%	87%	(+)	89%
Solanum pennellii	96%	84%	(+)	90%
Solanum chilense	95%	88%	(+)	90%
Solanum etuberosum	97%	79%	(+)	97%
Solanum palustre	92%	80%	(+)	97%
Solanum americanum	91%	75%	(+)	73%
Solanum melongena (eggplant)	89%	71%	(+)	82%
Solanum andreanum	97%	92%	(+)	98%
Solanum lignicaule	99%	93%	(+)	93%
Solanum vernei	98%	94%	(+)	96%
Iochroma cyaneum	90%	75%	(+)	74%
Capsicum annuum	87%	70%	(+)	79%
Nicotiana tabacum	84%	–	(–)	–
Petunia inflata	83%	–	(–)	–
Coffea humblotiana	79%	–	(–)	–
Arabidopsis thaliana	58%	–	(–)	–
Cucumis sativus	64%	–	(–)	–
Glycine max	63%	–	(–)	–

Supplemental Figure S8. Evolution of the VINV protein and enhancer *VInv*In2En. All numbers above the lines indicate the time (MYA) of species differentiation. (Supports Figure 6)
(**A**) Sequence similarities of the VINV proteins from different species in comparison to the VINV from RH potato.
(**B**) Similarities of intron 2 sequence in comparison to intron 2 from RH potato.
(**C**) Presence (+) and absence (-) of sequences homologous to the 200-bp *VInv*In2En of RH potato.
(**D**) Similarity of sequences homologous to the 200-bp *VInv*In2En of RH potato. Blue letters indicate tuber-bearing species; turquoise letters indicate non-tuber-bearing *Solanum* species closely related to potato, including tomato; red letters indicate species distantly related to potato.

```
Tomato_VInvIn2En ATAATTGGGATTAACATGAGATCTT--------GTGGCAGTAATGTTTTTTGCTTTTGTGCAATTTTCCAAT  64
M6_VInvIn2En     ATAATTGGGATTAACATGAGATCGTCTGATCGTGTGACAGCAATGTTTTTTGGTTTTGGGTAATTTTCCAAT  72
H28-7_VInvIn2En  ATAATTGGGATTAACATGAGATCGTCTGATCGTGTGACAGCAATGTTTTTTGGTTTTGGGTAATTTTCCAAT  72
RH_VInvIn2En     ATAATTGGGA--------TTAACATGAGATCGTGTGGCAGCAAAGTTTTTTGGTTTTGGGTAATTTTCCAAT  64
DM_VInvIn2En     ATAATTGGGA--------TTAACATGAGATCGTGTGGCAGCAAAGTTTTTTGGTTTTGGGTAATTTTCCAAT  64
                 **********          * * *        *** *** ** ******** ***** * ***********

Tomato_VInvIn2En AAAAAGAAAACACTTGATTGGGTCAGTATTATACAAGTTTGGAAACCAATCACGTTATGTGGGTCATACTTT  136
M6_VInvIn2En     AAAAATTAAACACATGATTGG-TCAGTTTTACACAAGTTTGGAAACCAATCACGTTATGTGGATCATACTTT  143
H28-7_VInvIn2En  AAAAATTAAACACATGATTGG-TCAGTTTTACACAAGTTTGGAAACCAATCACGTTATGTGGATCATACTTT  143
RH_VInvIn2En     AAAAATTAAACACATGATTGG-TCAGTTTTATACAAGTTTGGAAATCAATCACGTTATGTGGGTCATACTTT  135
DM_VInvIn2En     AAAAATTAAACACATGATTGG-CCAGTTTTATACAAGTTTGGAAATCAATCACGTTATGTGGGTCATACTTT  135
                 *****  ****** *******  **** *** ************* **************** *********

Tomato_VInvIn2En TTTGTAGTAATGTAATAATACCAATAGTGGGGCCCCCACTCAAAGTA---ATCCATC-TTCCACT         197
M6_VInvIn2En     TTTGTAGTAATGTAATAATTCCATTAGTTGGGCCCCCACCCAAATTATTTATCCATCTTTCCACT         208
H28-7_VInvIn2En  TTTGTAGTAATGTAATAATTCCATTAGTTGGGCCCCCACCCAAATTATTTATCCATCTTTCCACT         208
RH_VInvIn2En     TTTGTAGTAATGTAATAATTCCATTAGTTGGCCCCCCATCCAAATTATTTGTCCATCTTTCCACT         200
DM_VInvIn2En     TTTGTAGTAATGTAATAATTCCATTAGTTGGCCCCCCATCCAAATTATTTGTCCATCTTTCCACT         200
                 ******************* *** **** ** ******  **** **    ****** *******
```

Supplemental Figure S9. Alignment of the *VInv*In2En sequence from tomato and four different diploid potato genotypes. (Supports Figure 3)
Four potato genotypes, including M6, H28-7, RH, and DM, were used in the analysis. CBF/NF-Y motifs are marked in red; TCP motifs are in purple; GATA motif is in blue. “*” indicates identical nucleotides conserved among all genotypes. Note: single nucleotide polymorphisms and small indels are observed throughout the *VInv*In2En sequence, including those associated with the DNA motifs.

Supplemental Table S1. Sequences and mutated nucleotides within the 200-bp *VInv*In2En.

Target motif	Position within *VInv*In2En (bp)	Original sequence (5'-3')	Mutated sequence (5'-3')
B3	74-81	ACACATGA	ACCCGTGA
bHLH	75-80	CACATG	CCACTG
	112-120	ATCACGTTA	ATCAGGTTA
CBF/NF-Y	60-64	CCAAT	CTTAT
	81-85	ATTGG	ATCCG
	109-113	TCAAT	TGAAT
TCP	121-132	TGTGGGTCATAC	TGTGGGTGAAAC
	162-172	GTTGGCCCCCC	GTTAAACCCCC
GATA	15-25	CATGAGATCGT	CATGAGTTCGT

Note: Green and blue letter(s) represent original and replacement base(s), respectively.

Supplemental Table S2. List of sgRNAs used in all CRISPR/Cas experiments.

sgRNA Name	sgRNA Sequence (5'-3')
1a	GATTGGAGGTAGTACTAATT
2a	TTAGACTAAGGATGAAATAG
3a	CACGACAAGTCAAATAGAGT
3b	TGGAAATCAATCACGTTATG
2b	TAATAGTAATTGCTTCTAAG
1b	TATTTAAATTTTAGGGGAGG
R1	GACTAAGGATGAAATAGGGG
R2	GGGATTAACATGAGATCGTG
R3	GGACAAATAATTTGGATGGG
R4	TATTTGTCCATCTTTCCACT

Supplemental Table S3. List of primers for GUS reporter, CRISPR/Cas-based genome editing, splicing, and Y1H.

Gene/Detection	Prime pair	Forward primer (5'-3')	Reverse primer (5'-3')	Annealing temperature	Product length
*VInv*In2-Enzyme	VIT-F6/R6	caccGTGAGTTCAAAGTTA ATTATCATCA	cggaattcCTGTTGACCAATTTATT	58°C	1339 bp
*VInv*In2R-Enzyme	VIT-F8/R8	caccCTGTTGACCAATTTATT	cggaattcGTGAGTTCAAAGTTA ATTATCATCA	58°C	1339 bp
m35S-Enzyme	m35S-F2/R	cggaattcCCCGCCAATATATC	GAGAAAAGGGTCCTAACCAAG	58°C	153 bp
Kan gene	*Kan*-F/R	CCAACGCTATGTCCTGATAG	TTTGTCAAGACCGACCTGTC	51°C	529 bp
eGFP-GUS	Egg-F3/R3	CATCAAGGTGAACT TCAAGATCCG	AGTTCATAGAGATAA CCTTCACCCG	60°C	996 bp
m35S	m35S-F1/R	CCCGCCAATATATCCTGTCA	GAGAAAAGGGTCCTAACCAAG	58°C	145 bp
35S	35sEn-F1/m35S-R	AAGATGCCTCTGCCGACA	GAGAAAAGGGTCCTAACCAAG	58°C	315 bp
35SR	35sEnR-F1/m35S-R	TGCGAAGGATAGTGGGATT	GAGAAAAGGGTCCTAACCAAG	58°C	310 bp
*VInv*In2 & m35S	VIT-F6/m35S-R	caccGTGAGTTCAAAGTTA ATTATCATCA	GAGAAAAGGGTCCTAACCAAG	58°C	1482 bp
*VInv*In2R & m35S	VIT-F8/m35S-R	caccCTGTTGACCAATTTATT	GAGAAAAGGGTCCTAACCAAG	58°C	1482 bp
Kan gene	*Kan*-F3/R3	CCGCTCAGAAGAACTCGTCA	CAGACAATCGGCTGCTCTGA	60°C	721 bp
Cas9 gene	*Cas*-F1/R1	CTTGCGCGTCAACACAGAAA	GTCTCCTGAGGATAGCGTGC	60°C	362 bp
Editing	*VInv*-Edit-F/R	TAGTCACAATTAATTCATG	GAAATTTATGTTATAT ATTTTTATCC	55°C	648 bp
Editing	*VInv*-mut-F1/R1	CCTTGGTATATACCTAATCCC	GCGGCAAAAAGATAACGTTC	58°C	888 bp
Splicing	Splicing-F/R	CTCCGCCTCCCATTAC	TCAGATAAGTTGGTGGG	55°C	612 bp
pGADT7	pGADT7-F/R	TAATACGACTCACTATAGG GCGAGCGCCGCCATG	GTGAACTTGCGGGGTTTT TCAGTATCTACGATT	64°C	514-2740 bp

Supplemental Table S4. Analyses of variance (ANOVA) for RT-qPCR data from the Katahdin CRISPR/Cas9 lines.

Source	SS	DF	MS	*F* test
Model	14181.95	8	1772.74	29.28***
Genotypes	3146.75	3	1048.92	17.33***
Temperatures	2881.82	1	2881.82	47.61***
Genotype × Temperature	3762.35	3	1254.12	20.72***
Error	968.56	16	60.53	
Total	15150.51	24		

***P < 0.001; SS, sum square; DF, degree of freedom; MS, mean square.

Supplemental Table S5. Sequence analysis of intron 2 and enhancer *VInv*In2En from different plant species.

Species	Presence and size of exon 2 (bp)	Size of intron 2 (bp)	Sequences homologous to the 1327-bp intron 2 of RH (bp)	Fragments*	Gap size between fragments (bp)	Sequence similarity of intron 2	Sequences homologous to the 200-bp *VInv*In2En of RH (bp)	Fragments**	CBF/ NF-Y motifs	TCP motifs	GATA motif	Gap size between fragments (bp)	Sequence similarity compared to *VInv*In2En of RH
Solanum multiinterruptum	9	1304	1304	1	-	92% (1232/1340)	193	1	+, +, +	+, +	+	-	97% (187/193)
Solanum piurae	9	1810	1293	2	517	90% (1220/1355)	194	1	-, +, +	+, +	-	-	98% (190/194)
Solanum pinnatisectum	9	1300	1300	1	-	93% (1238/1335)	200	1	+, +, +	+, +	+	-	95% (190/200)
Solanum chomatophilum	9	1306	1306	1	-	93% (1250/1345)	200	1	+, +, +	+, +	+	-	97% (193/198)
Solanum morelliforme	9	1323	1323	1	-	94% (1248/1332)	199	1	+, +, +	+, +	+	-	95% (190/200)
Solanum jamesii	9	1318	1318	1	-	93% (1251/1342)	200	1	+, +, +	+, +	+	-	96% (191/200)
Solanum bulbocastanum	9	1320	1320	1	-	93% (1243/1342)	200	1	-, +, +	+, +	+	-	94% (188/200)
Solanum chacoense	9	1314	1314	1	-	93% (1256/1350)	208	1	+, +, +	+, +	+	-	92% (192/208)
Solanum brevicaule	9	1307	1307	1	-	93% (1246/1347)	207	1	+, +, +	+, +	+	-	92% (191/207)
Solanum candolleanum	9	1333	1333	1	-	94% (1274/1349)	208	1	+, +, +	+, +	-	-	92% (191/208)
Solanum tuberosum (RH)	9	1327	1327	1	-	100% (1327/1327)	200	1	+, +, +	+, +	+	-	100% (200/200)
Solanum tuberosum (DM)	9	1326	1326	1	-	99% (1325/1327)	200	1	+, +, +	+, +	+	-	99% (199/200)
Solanum commersonii	9	1611	1304	2	307	92% (1244/1348)	208	2	+, +, +	+, +	+	307	90% (189/209)
Solanum lycopersicum (tomato)	9	1377	1146	2	231	86% (1026/1189)	197	1	+, +, +	+, +	+	-	88% (177/201)

Solanum pimpinellifolium	9	1367	1145	2	222	87% (1028/1187)	197	1	+, +, +	+, +	+	-	89% (178/201)
Solanum pennellii	9	1392	1139	2	253	84% (1035/1233)	197	1	+, +, +	+, +	+	-	90% (181/201)
Solanum chilense	9	1354	1094	2	260	88% (993/1128)	196	1	+, +, +	+, +	+	-	90% (180/201)
Solanum etuberosum	9	1154	1154	1	-	79% (1068/1349)	200	1	-, +, +	+, +	+	-	97% (194/200)
Solanum palustre	9	1158	1158	1	-	80% (1075/1349)	200	1	-, +, +	+, +	+	-	97% (194/200)
Solanum americanum (American black nightshade)	9	2046	1129	6	12-449	75% (880/1171)	175	2	-, -, +	-, -	-	12	73% (128/176)
Solanum melongena (Eggplant)	9	1590	552	3	52-984	71% (447/633)	161	1	-, -, +	+, +	-	-	82% (135/164)
Solanum andreanum	9	2997	1298	3	204-1495	92% (1236/1337)	199	1	+, +, +	+, +	+	-	98% (195/200)
Solanum lignicaule	9	1515	1311	2	204	93% (1255/1352)	208	2	+, +, +	+, +	+	204	93% (193/208)
Solanum vernei	9	1309	1309	1	-	94% (1258/1340)	200	1	+, +, +	+, +	+	-	96% (191/200)
Iochroma cyaneum	9	2330	849	5	139-991	75% (695/921)	183	1	-, +, -	+, +	+	-	74% (154/209)
Capsicum annuum	-	4472	683	4	154-1910	70% (509/726)	153	1	-, +, -	+, -	-	-	79% (128/163)
Nicotiana tabacum	-	1256	-	-	-	-	-	-				-	-
Petunia inflata	-	1615	-	-	-	-	-	-				-	-
Coffea humblotiana	9	1978	-	-	-	-	-	-				-	-
Arabidopsis thaliana	9	780	-	-	-	-	-	-				-	-
Cucumis sativus	9	2210	-	-	-	-	-	-				-	-
Glycine max	9	1009	-	-	-	-	-	-				-	-

* Fragments: the number of sequence fragments within intron 2, which are homologous to the intron 2 of RH.

** Fragments: the number of sequence fragments within the region homologous to *VInv*In2En of RH.

Supplemental Table S6. Mutants and transgenic lines.

Mutant or transgenic line	Description
Potato Katahdin transgenic lines with m35S, 35S, *VInv*In2, and *VInv*In2R.	Four classes of transgenic lines were generated from *Agrobacterium*-mediated transformation in potato variety Katahdin by using constructs m35S-GUS, 35S-GUS, *VInv*In2-m35S-GUS, and *VInv*In2R-m35S-GUS, respectively.
Arabidopsis Col-0 transgenic lines with m35S, 35S, *VInv*In2, and *VInv*In2R.	Four classes of transgenic lines were generated from *Agrobacterium*-mediated transformation in Arabidopsis ecotype Col-0 by using constructs m35S-GUS, 35S-GUS, *VInv*In2-m35S-GUS, and *VInv*In2R-m35S-GUS, respectively.
Arabidopsis Col-0 transgenic lines #1 to #24.	A total of 24 classes of transgenic lines were generated from *Agrobacterium*-mediated transformation in Arabidopsis ecotype Col-0 by using constructs #1-m35S-GUS to #24-m35S-GUS, respectively.
Potato Katahdin transgenic lines with *VInv*In2En (Line #1, Line #2, and Line #3).	Three independent transgenic lines were generated from *Agrobacterium*-mediated transformation in potato variety Katahdin by using construct *VInv*In2En-m35S-GUS.
Arabidopsis Col-0 transgenic lines *VInv*In2En, B3, bHLH, CBF/NF-Y, TCP, and GATA.	Six classes of transgenic lines were generated from *Agrobacterium*-mediated transformation in Arabidopsis ecotype Col-0 by using constructs *VInv*In2En-m35S-GUS and *VInv*In2En-m35S-GUS with mutated motifs related to B3, bHLH, CBF/NF-Y, TCP, and GATA, respectively.
Potato DMF5-73-1 CRISPR/Cas9 deletion lines 2-2-8, 13-1-3, and 13-2-1.	Three independent homozygous CRISPR/Cas9 deletion lines were generated from *Agrobacterium*-mediated transformation in diploid potato clone DMF5-73-1 by using the Csy4-based CRISPR/Cas9 system.
Potato Katahdin T0 CRISPR/Cas9 lines KV78, KV87, and KV108.	Three independent T0 CRISPR/Cas9 line were generated from *Agrobacterium*-mediated transformation in potato cultivar Katahdin by using the U3/U6-based CRISPR/Cas9 system.
