# Supplementary material for: Molecular dissection of an intronic enhancer governing cold-induced expression of the vacuolar invertase gene in potato
Source: Plant Cell. 2024 Feb 20;36(5):1985–99. doi: 10.1093/plcell/koae050 (PMC11062429; doi:10.1093/plcell/koae050)
Supplement: koae050_Supplementary_Data [file koae050_supplementary_data.zip › tpc.00789.2023-s04.pdf]

THE PLANT CELL

Molecular dissection of an intronic enhancer governing cold-induced expression of the vacuolar invertase gene in potato

Xiaobiao Zhu, Airu Chen, Nathaniel Butler, Zixian Zeng, Haoyang Xin, Lixia Wang, Zhaoyan Lv, Dani Eshel, David Douches, and Jiming Jiang

Review Timeline:

Submission Date:	23-Aug-2023
Editorial Decision:	22-Oct-2023
Revision Received:	20-Nov-2023
Editorial Decision:	14-Dec-2023
Revision Received:	12-Jan-2024
Accepted:	16-Jan-2024

Dr. Jiming Jiang
Michigan State University
East Lansing, Michigan 48824

Dear Jiming / Dr. Jiang:

We have received reviews of your manuscript entitled "Molecular dissection of an intronic enhancer governing cold-induced expression of the vacuolar invertase gene in potato." Thank you for submitting your best work to The Plant Cell. The editorial board agrees that the work you describe is substantive, falls within the scope of the journal, and may become acceptable for publication, pending revision and potential re-review.

The study was found to be of strong interest and well-presented overall. Reviewers offer constructive criticisms and quite a few helpful suggestions from a variety of perspectives, which can be addressed point-by-point in revision and response. There were two areas of greatest concern to reviewers, and we ask you to pay special attention to the following points in preparing your revision:

1. The lack of evidence for actual TF binding was generally viewed as the largest concern; if binding data can be provided in the revision, this would be well-received. However, if it isn't feasible to provide binding data in support, it's important to acknowledge this point straightforwardly in the text.

2. A second substantial concern was the lack of data and/or discussion regarding the enhancer in CIS-resistant varieties. Any possible analysis in this direction would be helpful. For example, are there existing sequences available in public databases from CIS-resistant varieties that the authors could use to make a comparison between CIS-susceptible and resistant varieties?

Please contact us if there are ambiguous comments or if you wish to discuss the revision.

Given the nature of the comments, we are offering you 60 days from when we have issued this decision to complete the revision. If a revision is not returned within this time frame, and if you have not been granted an extension, we will withdraw the manuscript, which will leave you free to submit the work elsewhere. If you need an extension, we encourage you to contact us at any point before the 60 days have passed.

When you are ready to submit the revised version, please upload a highlighted copy that indicates all changes made in response to the editor and reviewer recommendations. Include an itemized list of all changes made in response to each of the reviewer's suggestions in the "Response to Reviewers" section; please note that reviewers do not have access to your cover letter, nor was this decision letter shared with them.

Thank you for the privilege of reviewing your work. We look forward to receiving your revised manuscript.

On behalf of the editorial board,

Molly Megraw, Board of Reviewing Editors
Robert Schmitz, Senior Editor
Blake Meyers, Editor-in-Chief

The Plant Cell

Please note the following:

-The Plant Cell now requires authors to complete and submit an author revisions checklist upon submission of a revised manuscript. The aim of the checklist is to aid authors in preparing a high-quality manuscript, facilitate the review and assessment of revised manuscripts, and help to ensure that journal standards are maintained across the board. If your manuscript is accepted, the completed checklist will be published as supplemental material attached to the article online. Please download a copy of the checklist (pdf fillable form) at this link, for submission with your revised manuscript: https://tpc.msubmit.net/html/Author_Revisions_Checklist.pdf.

-Supplemental materials should be restricted to large datasets and tables, presentation of replicates, and validation of reagents, methods, or genotypes. Any data that are used to support the major claims must be in the main manuscript. Supplemental figure legends must indicate what figure in the main manuscript is supported by the supplemental data presented. Please justify how each of the supplemental figures meet the criteria.

-Sampling methods and nature of "biological replicates" should be described precisely (i.e. different plants, parts of

plants, pooled tissue, independent pools of tissue, sampled at different times, etc), along with a clear description of and rationale for any statistical analyses conducted. The reader should know exactly what was sampled; what forms the basis of the calculation of any means and statistical parameters reported. This is also necessary to ensure that proper statistical analysis was conducted.

-Want to add this revision deadline to your calendar? Click below!

--- Reviewer comments:

Reviewer #1 (Comments for the Author):

Importance of finding: This paper characterizes the molecular mechanism that controls the expression of the vacuolar invertase (VInv) gene in response to cold temperature in potato. Specifically, the authors found that a 200-bp enhancer located in the second intron of VInv is responsible for the cold-induced response of VInv. This is important because the increased expression of VInv at cold storage temperature triggers cold-induced sweetening (CIS) in CIS-susceptible potato genotypes. CIS is the accumulation of reducing sugars in tubers, which has a negative effect on the processing qualities of potato.

Quality of experiments: The experiments are scientifically sound and well described. The authors provided quality control for all the data they presented. I found the use of DMF5-73-1 (self-compatible diploid clone) for CRISPR-based gene editing very interesting. This clone should be very useful for the potato research community.

Points in favor: This is quite an interesting regulation mechanism, i.e. the enhancer is located in an intron.

Points detracting: None that would preclude publication

Author actions necessary for acceptance: I am curious to know whether the 200-bp enhancer is also present in CIS-resistant varieties. If it is, how does its sequence differ between susceptible and resistant varieties? Can SNPs in this region explain the differences of susceptibility? The authors should add this aspect in the discussion.

Minor points:
Introduction:
Lines 74-76: The timeline seems to ignore earlier work mentioned later in the introduction, e.g. Zrenner et al 1996. I suggest the authors rephrase this section to better reflect the history of VInv and CIS.
Line 85: repetitive of lines 78-79

Results:
Lines 113-114: specify which potato genotype was transformed (Katahdin I believe based on the methods section, but this should be specified here so that the lack of a mini35S control in Figure 2F is not an issue).

Figure S4: typo: edit Cyy4 to Csy4

Discussion:
Line 288: edit typo 'tomtao'
Line 305: edit typo 'osmatic'

Methods:
Line 365: edit typo 'wes'

Author contributions: I assume that you meant D.E. and not D.S.

References:
The abbreviation of the Journal of Agricultural and Food Chemistry is J Agric Food Chem

Reviewer #2 (Comments for the Author):

The authors of "Molecular dissection of an intronic enhancer governing cold-induced expression of the vacuolar invertase gene in Potato" describe a newly discovered Enhancer region with an important function during the cold-

response of potato tubers. The "cold-induced sweetening" (CIS) is a major postharvest concern for potatoes as prolonged cold temperatures lead to an accumulation of reduced sugars and sweetening of potato tubers. VLnv is one of the major genes involved in CIS, however, the cis-elements responsible for the cold regulation of VLnv were not known (except that the ATG upstream promoter was not sufficient for the cold regulation of VLnv). The authors identified an accessible chromatin region in the second intron and rigorously tested the enhancer function of this candidate region. Using GUS-fusion, heterologous expression, and CRISPR-editing the authors could convincingly establish the necessary role of a 200bp core enhancer VLnvIn2EN (2En) for the normal cold-response of VLnv also in potato. Furthermore, the authors demonstrated that editing any of at least 3 motifs disrupts cold-induction of VLnv. While it remains unclear which exact TF binding (CBF/NF-Y, TCP, and GATA motifs) and if indeed the triple binding of a TF complex is needed or sufficient for cold induction, the evidence is rather convincing as to the important function of this evolutionary conserved @en cis-elements.

Overall I think the paper presents the evidence well in a very concise manner. While there are always more questions that could be answered, I believe the study stands well on its own and responds with a detailed example of an important cis-element an intron. I therefore would only ask for a few minor revisions specifically of the discussion and 1 minor addition of additional controls to a qPCR experiment.

Minor points:
1) Lines 247-249: Two things i) I think the description of the expression of VLnv was reduced is not optimal. I guess the expected cold-induced induction of VLnv transcripts is 94.4%, 83.6%, and 72.7% lower compared to KV. ii) I write expected because the three lines need to be checked under 22C. This is one instance where I believe expanding the qPCR to include an accurate comparison of the three lines under 22C is simply the better control.

2) The last paragraph of the discussion in my opinion overstates a few things that are not really needed I think as the otherwise strong evidence speaks for itself. i) There are also still many important questions remaining to solve the puzzle. Which TFs are targeting the 2En? Why do potato ecotypes with solid differences in CIS have identical 2En sequences? Are there other enhancers in those ecotypes? Is the 2En region accessible in those ecotypes? What's the reason for the tuber-specific cold response of VLNC? Is the 2En not accessible in other tissues? If it is also bound in other tissues what's the reason? And I could name many more. If the authors insist on their statement most of these questions should be answered, which is maybe not worth the statement.

ii) Similarly, I think the statement of this being the first multiple TF enhancers may not be accurate. There is for example the PIF4/ARF6/BRZ1 TF module which binds enhancers together, at separate motifs. In fact, I believe that module was found because of the close proximity clustering of the TF motifs. IMHO there are other cases where we know that close proximity multiple TF motifs clustering to controlling a mRNA. Maybe not for cold stress but again I feel it's an unnecessary overstatement.

3) I think the discussion should be expanded a bit. There is very little to the TF, their potential interaction, and known functions in cold response. Is this combination of the 3 motifs elsewhere found in the genome? Do the families of TFs play known roles in cold response and are they known to interact? I understand that the exact TFs are not known.

4) Line 754: The common and in the original paper defined term is minimal 35S or m35S and should be kept instead of mini35S

Reviewer #3 (Comments for the Author):

In this manuscript authored by Zhu et al., the authors detail their discovery of a 200-base pair transcriptional enhancer in the intron 2 region of VInv named VInvIn2En that plays a crucial role in regulating the cold-induced expression of the VInv gene. To investigate its function, the authors employed CRISPR/Cas9-mediated genome editing technology to generate VInvIn2En deletion lines, which exhibited a significant reduction in VInv transcription levels and also reduced cold-induced sweetening (CIS) in potato tuber during cold storage. However, the manuscript does not address the underlying mechanism by which VInvIn2En responds to cold stress

Several improvements are necessary for this manuscript to reach a publishable standard. I have provided a series of comments and suggestions below to assist the authors in addressing these issues.

1. Several potato cultivars were used in this study, but only a few were addressed their features, such as DMF5-73-1 is ideal for CRISPR/Cas experiments, and Katahdin is highly sensitive to CIS. Please also offer brief background introduction for others, such as RH and DM1-3.
2. It is not clear where to get the conclusion that enhancer is located within 475-bp (line 145) .
3. Vacuolar invertases play essential roles in cell expansion and sugar accumulation, which are related to plant growth

and development. Therefore, silencing of the vacuolar invertase gene can cause major developmental defects in plants. (line 285-288). What are phenotypes VInvIn2En deletion lines during vegetative growth in this study?

Editor comments:

We noticed that in a few places (Figures 2A, 4F, 5) your figures utilize a serif font (Times, I think) and/or mix font types, and the journal requirement is a sans serif font such as Arial. This is described in our instructions to authors. As you prepare the next version of your figures, I would ask that you please change the font throughout your figures to Arial. Please check your supplemental figures too.

Response to comments from Reviewer #1:

Importance of finding: This paper characterizes the molecular mechanism that controls the expression of the vacuolar invertase (VInv) gene in response to cold temperature in potato. Specifically, the authors found that a 200-bp enhancer located in the second intron of VInv is responsible for the cold-induced response of VInv. This is important because the increased expression of VInv at cold storage temperature triggers cold-induced sweetening (CIS) in CIS-susceptible potato genotypes. CIS is the accumulation of reducing sugars in tubers, which has a negative effect on the processing qualities of potato.

Quality of experiments: The experiments are scientifically sound and well described. The authors provided quality control for all the data they presented. I found the use of DMF5-73-1 (self-compatible diploid clone) for CRISPR-based gene editing very interesting. This clone should be very useful for the potato research community.

Points in favor: This is quite an interesting regulation mechanism, i.e. the enhancer is located in an intron.

Points detracting: None that would preclude publication

Response: We are thankful to the Reviewer for the positive comments on this work.

Author actions necessary for acceptance: I am curious to know whether the 200-bp enhancer is also present in CIS-resistant varieties. If it is, how does its sequence differ between susceptible and resistant varieties? Can SNPs in this region explain the differences of susceptibility? The authors should add this aspect in the discussion.

Response: Overall, the sequence of the 200-bp *VInv*In2En enhancer is highly conserved among different potato genotypes. For example, DM and RH potato lines have significantly different levels of CIS resistance despite having identical *VInv*In2En sequences. Thus, we believe that post-transcriptional regulation of the VINV protein plays a role in CIS as discussed in the manuscript.

Following the Reviewer's comments, we examined the *VInv*In2En sequence in additional potato genotypes, including the diploid clone H28-7 that is highly resistant to CIS (Bhaskar et al. 2010 Plant Physiology). We did find sequence polymorphism, including SNPs within the GATA and TCP motifs. Therefore, we cannot exclude the possibility that sequence polymorphism of the *VInv*In2En sequence also plays a role of CIS resistance in different potato genotypes. We have added this analysis in the revised manuscript, including a supplemental figure (Figure S9) illustrating the polymorphism of the *VInv*In2En sequence.

Minor points:
Introduction:

Lines 74-76: The timeline seems to ignore earlier work mentioned later in the introduction, e.g. Zrenner et al 1996. I suggest the authors rephrase this section to better reflect the history of VInv and CIS.

Response: We have rephrased the text in this paragraph to better describe the historical research on the *VInv* gene in CIS.

Line 85: repetitive of lines 78-79

Response: The first sentence has been removed.

Results:
Lines 113-114: specify which potato genotype was transformed (Katahdin I believe based on the methods section, but this should be specified here so that the lack of a mini35S control in Figure 2F is not an issue).

Response: Thank you for pointing this out, you are correct. We have added that Katahdin was used for transformation in the revised manuscript.

Figure S4: typo: edit Cyy4 to Csy4
Discussion:
Line 288: edit typo 'tomtao'
Line 305: edit typo 'osmatic'
Methods:
Line 365: edit typo 'wes'
Author contributions: I assume that you meant D.E. and not D.S.

Response: The typos are fixed. Thank you for careful reading of the manuscript.

References:
The abbreviation of the Journal of Agricultural and Food Chemistry is J Agric Food Chem

Response: The abbreviation was fixed.

Response to comments from Reviewer #2:

The authors of "Molecular dissection of an intronic enhancer governing cold-induced expression of the vacuolar invertase gene in Potato" describe a newly discovered Enhancer region with an important function during the cold-response of potato tubers. The "cold-induced sweetening" (CIS) is a major postharvest concern for potatoes as prolonged cold temperatures lead to an

accumulation of reduced sugars and sweetening of potato tubers. VLnv is one of the major genes involved in CIS, however, the cis-elements responsible for the cold regulation of VLnv were not known (except that the ATG upstream promoter was not sufficient for the cold regulation of VLnv). The authors identified an accessible chromatin region in the second intron and rigorously tested the enhancer function of this candidate region. Using GUS-fusion, heterologous expression, and CRISPR-editing the authors could convincingly establish the necessary role of a 200bp core enhancer VLnvIn2EN (2En) for the normal cold-response of VLnv also in potato. Furthermore, the authors demonstrated that editing any of at least 3 motifs disrupts cold-induction of VLnv. While it remains unclear which exact TF binding (CBF/NF-Y, TCP, and GATA motifs) and if indeed the triple binding of a TF complex is needed or sufficient for cold induction, the evidence is rather convincing as to the important function of this evolutionary conserved @en cis-elements.

Response: We appreciate the comments. We have conducted yeast one-hybrid assays to identify proteins interacting with the *VInv*In2En enhancer. Two CBF/NF-Y proteins, StNF-YC1 and StNF-YC9, were identified in the assays, which validate the function of the CBF/NF-Y motifs identified in the *VInv*In2En enhancer (**Figure I**). We have added this new result in the revised manuscript, including the figure below (new Figure 4 in the revised manuscript).

We agree with the Reviewer that our current data does not conclude whether the triple binding of a TF complex is needed or sufficient for cold induction. We intend to address this question in potato, which is one of our future research goals.

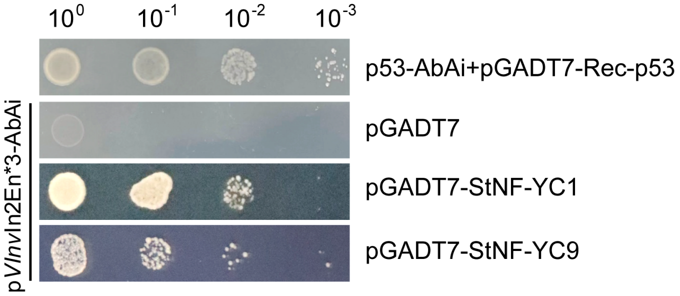

Figure I. Identification of StNF-YC1 and StNF-YC9 proteins that bind to *VInv*In2En using Y1H assay. The pGADT7 vector was used as negative control, and a combination of two constructs (p53-AbAi and pGADT7-Rec-p53) were used as positive control.

Overall I think the paper presents the evidence well in a very concise manner. While there are always more questions that could be answered, I believe the study stands well on its own and responds with a detailed example of an important cis-element an intron. I therefore would only ask for a few minor revisions specifically of the discussion and 1 minor addition of additional controls to a qPCR experiment.

Response: We are thankful to the Reviewer's conclusion, to which we fully agree. We have added the requested additional control for the qPCR analysis (see below).

Minor points:

1) Lines 247-249: Two things i) I think the description of the expression of VLnv was reduced is

not optimal. I guess the expected cold-induced induction of VLnv transcripts is 94.4%, 83.6%, and 72.7% lower compared to KV. ii) I write expected because the three lines need to be checked under 22C. This is one instance where I believe expanding the qPCR to include an accurate comparison of the three lines under 22C is simply the better control.

Response: **(i)** We have modified the description of the expression of *VInv* in the revised manuscript. The expression level of *VInv* in cold-stored (4°C) tubers of the three CRISPR/Cas lines (KV78, KV87, and KV108) was 6.6%, 16.4%, and 27.3%, respectively, of the wild-type Katahdin. **(ii)** We have added the expression data of the three CRISPR/Cas lines under 22°C (**Figure II**), which is used to replace the original Figure 4G.

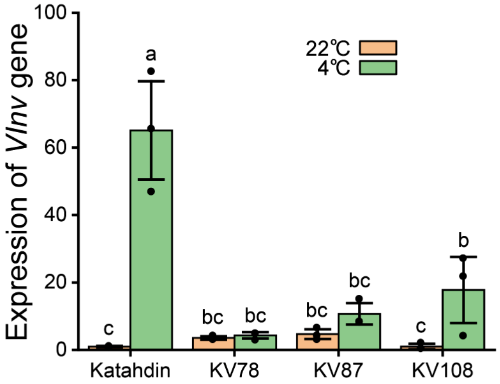

Figure II. qRT-PCR-based analysis of *VInv* expression relative to the *Actin97* gene of the three CRISPR/Cas lines. Expression was analyzed using tubers after 2 weeks of storage at 22°C and 4°C, respectively. The *y* axis represents the relative expression level normalized by setting *VInv* expression in 22°C-stored tubers of the wild-type Katahdin to 1. Bars represent mean ± standard deviation of three independent tuber samples. Different lowercase letters represent statistically significant differences at the 0.05 level.

2) The last paragraph of the discussion in my opinion overstates a few things that are not really needed I think as the otherwise strong evidence speaks for itself. i) There are also still many important questions remaining to solve the puzzle. Which TFs are targeting the 2En? Why do potato ecotypes with solid differences in CIS have identical 2En sequences? Are there other enhancers in those ecotypes? Is the 2En region accessible in those ecotypes? What's the reason for the tuber-specific cold response of VLNC? Is the 2En not accessible in other tissues? If it is also bound in other tissues what's the reason? And I could name many more. If the authors insist on their statement most of these questions should be answered, which is maybe not worth the statement.

Response: We thank the Reviewer for the fair comment. The questions raised by the Reviewer are scientifically sound and we intend to pursue the answers for these questions in the future. We have removed this entire paragraph in the revised manuscript.

ii) Similarly, I think the statement of this being the first multiple TF enhancers may not be accurate. There is for example the PIF4/ARF6/BRZ1 TF module which binds enhancers

together, at separate motifs. In fact, I believe that module was found because of the close proximity clustering of the TF motifs. IMHO there are other cases where we know that close proximity multiple TF motifs clustering to controlling a mRNA. Maybe not for cold stress but again I feel it's an unnecessary overstatement.

Response: The statement was removed.

3) I think the discussion should be expanded a bit. There is very little to the TF, their potential interaction, and known functions in cold response. Is this combination of the 3 motifs elsewhere found in the genome? Do the families of TFs play known roles in cold response and are they known to interact? I understand that the exact TFs are not known.

Response: We thank for this comment and have added a paragraph to discuss the potential TFs involved in transcriptional regulation mediated by the *VInv*In2En enhancer.

Following the Reviewer's suggestion we searched all potato DHSs that contain a combination of all three DNA motifs (CBF/NF-Y, TCP and GATA) identified in *VInv*In2En. We found only two additional DHSs with at least one copy of each of the three motifs (with minor sequence variation). The two putative cognate genes of these two DHSs are either annotated as "unknown function" or not associated with abiotic stress response.

4) Line 754: The common and in the original paper defined term is minimal 35S or m35S and should be kept instead of mini35S

Response: We have replaced every "mini35S" with "m35S".

Response to comments from Reviewer #3:

In this manuscript authored by Zhu et al., the authors detail their discovery of a 200-base pair transcriptional enhancer in the intron 2 region of VInv named VInvIn2En that plays a crucial role in regulating the cold-induced expression of the VInv gene. To investigate its function, the authors employed CRISPR/Cas9-mediated genome editing technology to generate VInvIn2En deletion lines, which exhibited a significant reduction in VInv transcription levels and also reduced cold-induced sweetening (CIS) in potato tuber during cold storage. However, the manuscript does not address the underlying mechanism by which VInvIn2En responds to cold stress.

Response: We respect the opinion from the Reviewer. It has taken us more then seven years to identify, validate, and dissect the function of *VInv*In2En as a transcriptional enhancer. We agree that many questions remain to be answered for a complete understanding of *VInv*In2En-mediated response to cold stress, which is also pointed out by Reviewer 2 (see the summary statement and

minor point #2 from Reviewer 2), but many of these questions are beyond the scope of the current study.

Several improvements are necessary for this manuscript to reach a publishable standard. I have provided a series of comments and suggestions below to assist the authors in addressing these issues.

1. Several potato cultivars were used in this study, but only a few were addressed their features, such as DMF5-73-1 is ideal for CRISPR/Cas experiments, and Katahdin is highly sensitive to CIS. Please also offer brief background introduction for others, such as RH and DM1-3.

Response: We have added brief descriptions for the RH and DM1-3 potato lines in the revised manuscript.

2. It is not clear where to get the conclusion that enhancer is located within 475-bp (line 145).

Response: Intron 2 of *VInv* is 1,327 bp long (0 to 1,327 bp). The 200-bp *VInv*In2En spans 678-877 bp in the intron; the 475-bp DHS spans 597-1071 bp in the intron. This information has been added to the revised manuscript.

3. Vacuolar invertases play essential roles in cell expansion and sugar accumulation, which are related to plant growth and development. Therefore, silencing of the vacuolar invertase gene can cause major developmental defects in plants. (line 285-288). What are phenotypes VInvIn2En deletion lines during vegetative growth in this study?

Response: We did not observe any unambiguous phenotypic changes from the three CRISPR/Cas lines developed from Katahdin. We previously developed RNAi-mediated *VInv* silencing lines of Katahdin. The silencing lines did not show phenotypic changes or yield losses despite a 99% reduction of *VInv* gene transcription (Bhaskar et al. 2010, Plant Physiology). Thus, it was not unexpected for us to see an unaltered phenotype for the Katahdin CRISPR/Cas lines. We hypothesize the *VInv* gene is adapted for a distinct role in tuber-bearing species for cold stress response, which is discussed in the Discussion section.

Dear Dr. Jiming Jiang:

We have received reviews of your manuscript entitled "Molecular dissection of an intronic enhancer governing cold-induced expression of the vacuolar invertase gene in potato." On the basis of the advice received, the board of reviewing editors would like to accept your manuscript for publication in The Plant Cell. This acceptance is contingent on revision based on the comments of our reviewers. In particular, please consider the following:

Beyond helpful typo corrections, Reviewer #1 provides two constructive requests for clarification: stating the selection criteria used to identify the 33 Y1H assay candidates, and if available an inclusion or discussion of data that would help correlate SNPs/small indels in VInvIn2En with gene expression. We'd like to ask that you address these modest requests (as possible) in a minor revision.

Please highlight all changes and include a detailed annotation of changes of the text, with line numbers, and noting your responses to the comments.

To submit your revised manuscript, click:

Link Not Available

If you have any questions about the revision submission procedures, please contact the Editorial Office Staff (tpc-submissions@aspb.org). If you cannot return the revised manuscript within 30 days, please let us know. Otherwise, we will assume that you have elected not to revise the manuscript and withdraw it.

Thank you very much for the privilege of reviewing this work. I look forward to receiving the next version.

On behalf of the editorial board,

Molly Megraw, Board of Reviewing Editors
Robert Schmitz, Senior Editor
Blake Meyers, Editor-in-Chief

The Plant Cell

Reviewer #1 (Comments for the Author):

Typos:
New Y1H paragraph in results: "candidate" -> "candidates"
New paragraph in results just before discussion: "it" and "polymorphium" -> "its" and "polymorphism", "comparision" -> "comparison"
New paragraph on TFs in discussion: "transcritioal" -> "transcriptional

It is not clear in the results or methods what criteria were used to narrow down to 33 candidates for the Y1H assay. Please explain.

Do the authors have any data (or are there any existing data in the literature) showing the transcript levels of VInv in response to cold temperatures in CIS-resistant clones M6 and H28-7? This would help correlate SNPs/small indels in VInvIn2En with gene expression.

Reviewer #2 (Comments for the Author):

Dear authors. I think this is a well-done revision. The added experiments benefit the hypotheses. I also agree with the changes to the manuscript text and have no further concerns.

Reviewer #3 (Comments for the Author):

I am pleased to acknowledge the significant improvements that have been incorporated in response to the earlier comments. The addition of Figure 4, which elucidates the Y1H assay results for the identification of StNF-YC1 and StNF-YC9 proteins

binding to VInvIn2En, provides substantial and convincing evidence supporting findings in this study. In light of these revisions and the thoroughness of responses to the review comments, I believe that the revised manuscript is now well-prepared for publication.

Please also note the following:

-The Plant Cell now requires authors to complete and submit an author revisions checklist upon submission of a revised manuscript. The aim of the checklist is to aid authors in preparing a high-quality manuscript, facilitate the review and assessment of revised manuscripts, and help to ensure that journal standards are maintained across the board. If your manuscript is accepted, the completed checklist will be published as supplemental material attached to the article online. Please download a copy of the checklist (pdf fillable form) at this link, for submission with your revised manuscript: https://tpc.msubmit.net/html/Author_Revisions_Checklist.pdf.

-Supplemental materials should be restricted to large datasets and tables, presentation of replicates, and validation of reagents, methods, or genotypes. Any data that are used to support the major claims must be in the main manuscript. Supplemental figure legends must indicate what figure in the main manuscript is supported by the supplemental data presented. Please justify how each of the supplemental figures meet the criteria.

-Sampling methods and the nature of "biological replicates" should be described precisely (i.e. different plants, parts of plants, pooled tissue, independent pools of tissue, sampled at different times, etc.), along with a clear description of and rationale for any statistical analyses conducted. The reader should know exactly what was sampled; what forms the basis of the calculation of any means and statistical parameters reported. This is also necessary to ensure that proper statistical analysis was conducted.

Response to comments from Reviewer #1:

Typos:
New Y1H paragraph in results: "candidate" -> "candidates"
New paragraph in results just before discussion: "it" and "polymorphium" -> "its" and "polymorphism", "comparision" -> "comparison"
New paragraph on TFs in discussion: "transcritioal" -> "transcriptional

Response: All typos have been corrected.

It is not clear in the results or methods what criteria were used to narrow down to 33 candidates for the Y1H assay. Please explain.

Response: We have added the information describing how the 33 candidate proteins were identified in both Results and Methods.

Do the authors have any data (or are there any existing data in the literature) showing the transcript levels of VInv in response to cold temperatures in CIS-resistant clones M6 and H28-7? This would help correlate SNPs/small indels in VInvIn2En with gene expression

Response: This is a very good comment/suggestion. *VInv* expression in cold-stored tubers is very low in CIS-resistant germplasm H28-7 (Bhaskar et al. 2010, Plant Physiology). We have added this information and relevant discussion on potential impact of sequence variation of VInvIn2En on CIS.

Dear Dr. Jiming Jiang:

We are pleased to inform you that your paper entitled "Molecular dissection of an intronic enhancer governing cold-induced expression of the vacuolar invertase gene in potato" has been accepted for publication in The Plant Cell, pending a final editorial review by a science editor. At this stage, your manuscript will be evaluated by a science editor with respect to its presentation of scientific content, compliance with journal policies, and presentation for a broad readership. The Plant Cell has contracted with Plant Editors (planteditors.com) to provide this service to our authors, and you will soon receive additional information on this process.

Please note that each author needs to link their ORCID identifier to their account in the system before your manuscript can be published. If any authors do not have an ORCID linked to their account, they will receive a message with a link to complete this task. Please ensure that ALL of your coauthors have completed this task as soon as possible.

ASPB offers an OPEN option that allows authors to have their online articles available for free to all users immediately upon publication. For more information about the ASPB OPEN option, refer to the Final Submission Checklist Form.

The Plant Cell and The Arabidopsis Information Resource (TAIR) are collaborating to collect functional annotation data about Arabidopsis genes from authors. This includes information about the gene's molecular function (e.g., kinase activity, ATP synthetase activity), the biological process/es it is involved in (e.g., endosperm development, threonine biosynthesis), its subcellular location (e.g., nucleus, ER), anatomical or developmental expression pattern (e.g., leaf, ovule, flower stage 10, seedling stage), or its partner in a protein-protein interaction (e.g., AT1G01010 interacts with AT1G01020).

If your paper contains results falling into one or more of these categories for Arabidopsis genes, we request that you now submit these data for inclusion in TAIR by filling in the form provided at the following URL: https://www.arabidopsis.org/doc/submit/functional_annotation/123. If you need further clarification on what types of data can be submitted please contact curator@arabidopsis.org.

Finally, we encourage your submission of artwork for the journal cover. Monthly issues will have an online cover image and selected covers will be used for posters, other promotional items, and "wallpaper" for mobile devices. For more information, refer to Cover Submission in the Instructions for Authors https://tpc.msubmit.net/cgi-bin/main.plex?form_type=display_auth_instructions.

We look forward to seeing your paper published.

On behalf of the editorial board,

Reviewing Editor, Board of Reviewing Editors
Robert Schmitz, Senior Editor
Blake Meyers, Editor-in-Chief

The Plant Cell

=====
IMPORTANT REMINDER: PEER REVIEW REPORTS
=====

If you opted to publish a peer review report along with your article during the original submission process, it will be prepared by the editorial staff and publicly posted with your manuscript, inside the zip file that contains any other supplemental material. As a reminder, the peer review report is a public record of all comments from editors and reviewers, as well as your prior responses, as you received them in the decision letters for each draft of your manuscript. If you agreed to publish this report and have changed your mind, or are not sure if you selected this option, please contact the editorial office as soon as possible before signing the license agreement from our publisher.

=====

----FOR ASPB OFFICE USE ONLY (DO NOT EDIT)----
MSID: 35843
Corresponding Author: Dr. Jiang
